# Candidate Biomarkers for the Prediction and Monitoring of Partial Remission in Pediatric Type 1 Diabetes

**DOI:** 10.3389/fimmu.2022.825426

**Published:** 2022-02-23

**Authors:** Laia Gomez-Muñoz, David Perna-Barrull, Josep M. Caroz-Armayones, Marta Murillo, Silvia Rodriguez-Fernandez, Aina Valls, Federico Vazquez, Jacobo Perez, Raquel Corripio, Luis Castaño, Joan Bel, Marta Vives-Pi

**Affiliations:** ^1^ Immunology Department, Germans Trias i Pujol Research Institute and University Hospital, Autonomous University of Barcelona, Badalona, Spain; ^2^ Department of Political and Social Sciences, Health Inequalities Research Group (GREDS-EMCONET), Pompeu Fabra University, Barcelona, Spain; ^3^ Johns Hopkins University–Pompeu Fabra University Public Policy Center, Barcelona, Spain; ^4^ Pediatrics Department, Germans Trias i Pujol Research Institute and University Hospital, Autonomous University of Barcelona, Badalona, Spain; ^5^ Endocrinology Department, Germans Trias i Pujol Research Institute and University Hospital, Autonomous University of Barcelona, Badalona, Spain; ^6^ Pediatric Endocrinology Department, Parc Taulí Hospital Universitari, Institut d’Investigació i Innovació Parc Taulí I3PT, Autonomous University of Barcelona, Sabadell, Spain; ^7^ Cruces University Hospital, Biocruces Bizkaia Research Institute, UPV/EHU, CIBERDEM, CIBERER, Endo-ERN, Bilbao, Spain

**Keywords:** type 1 diabetes (T1D), partial remission phase, honeymoon, biomarkers, pediatrics, prediction model, immune cell subpopulations, autoimmunity

## Abstract

The partial remission (PR) phase, a period experienced by most patients with type 1 diabetes (T1D) soon after diagnosis, is characterized by low insulin requirements and improved glycemic control. Given the great potential of this phase as a therapeutic window for immunotherapies because of its association with immunoregulatory mechanisms and β-cell protection, our objective was to find peripheral immunological biomarkers for its better characterization, monitoring, and prediction. The longitudinal follow-up of 17 pediatric patients with new-onset T1D over one year revealed that, during the PR phase, remitter patients show increased percentages of effector memory (EM) T lymphocytes, terminally differentiated EM T lymphocytes, and neutrophils in comparison to non-remitter patients. On the contrary, remitter patients showed lower percentages of naïve T lymphocytes, regulatory T cells (T_REG_), and dendritic cells (DCs). After a year of follow-up, these patients also presented increased levels of regulatory B cells and transitional T1 B lymphocytes. On the other hand, although none of the analyzed cytokines (IL-2, IL-6, TGF-β1, IL-17A, and IL-10) could distinguish or predict remission, IL-17A was increased at T1D diagnosis in comparison to control subjects, and remitter patients tended to maintain lower levels of this cytokine than non-remitters. Therefore, these potential monitoring immunological biomarkers of PR support that this stage is governed by both metabolic and immunological factors and suggest immunoregulatory attempts during this phase. Furthermore, since the percentage of T_REG_, monocytes, and DCs, and the total daily insulin dose at diagnosis were found to be predictors of the PR phase, we next created an index-based predictive model comprising those immune cell percentages that could potentially predict remission at T1D onset. Although our preliminary study needs further validation, these candidate biomarkers could be useful for the immunological characterization of the PR phase, the stratification of patients with better disease prognosis, and a more personalized therapeutic management.

## Introduction

Type 1 diabetes (T1D) is an autoimmune T-cell-mediated disease against pancreatic β-cells, which leads to the insufficient production of insulin and overt hyperglycemia (1). This chronic metabolic disease affects a growing number of children and adolescents (2), and because of the β-cell function decay, patients rely on exogenous insulin therapy for life, which only delays the appearance of long-term secondary complications. Although its exact etiology and pathogenesis are still elusive, the development of T1D involves complex interactions between β-cells and innate and adaptive immune cells, namely, T and B lymphocytes, NK cells, monocytes, dendritic cells (DCs), and neutrophils (3). Different strategies capable of stopping the autoimmune process and promoting β-cell recovery have been developed in experimental models, but none of them has achieved total remission in humans or managed to prevent or cure the disease (4, 5). This failure could be partially explained by the lack of an optimal checkpoint for immune interventions, and the insufficient biomarkers for the proper stratification of patients, an unmet need in clinical trials (6, 7).

As for this last point, shortly after diagnosis and the initiation of insulin therapy, between 50 and 80% of patients with T1D experience a transient partial remission (PR) period, also called the honeymoon phase, which is marked by low requirements of exogenous insulin and diminished glycated hemoglobin (HbA1c) levels (8). This stage, characterized by metabolic and immunological alterations, could constitute a unique window for therapeutic interventions. This period can last from weeks to years with an average of 9 months of duration (9), but in some uncommon cases, a complete or long-lasting remission has been described (10, 11). Patients with this improved glucose control may be at a lower risk for the short- and long-term complications of T1D, such as chronic microvascular complications.

Unfortunately, the mechanisms underlying the PR phase are poorly characterized, but this natural phenomenon has been attributed to both the reduction of glucose toxicity after the initiation of the insulin therapy and the consequent β-cell rest, recovery, and regeneration with improved endogenous insulin production (12). At a clinical level, bicarbonate concentrations >15 mg/dl, age >5 years, male sex, higher body mass index (BMI) values, lower HbA1c levels, and <3 diabetes-associated autoantibodies can predispose to PR in children and adolescents with new-onset T1D (13, 14). Furthermore, mechanisms of immune regulation are non-linear along the natural history of T1D—matching well with the proposed relapsing-remitting character of the disease course—and the PR phase is associated with these immunomodulatory changes (15, 16). Consistent with this idea, studies investigating changes in immune parameters have reported that patients with the highest frequency of CD4^+^CD25^+^CD127^hi^ lymphocytes at disease onset experience the longest PR (17, 18), that increased levels of regulatory T, B, and NK cells can be found during T1D progression (which could reflect attempts at restoring self-tolerance) (19, 20), and that the absence of IL-4, TNF-α, IL-10, and IL-13 in sera correlates with the length of the remission period (21). Interestingly, peripheral antigen-specific regulatory T cells (T_REG_) diminish during the honeymoon phase in comparison to the time of diagnosis (22). Nevertheless, few studies have compared patients with and without PR to find specific and reliable biomarkers of this phase.

Non-invasive approaches like the use of peripheral blood samples are essential to identify biomarkers of T1D progression and remission. Since many studies have shown that the progression of T1D is associated with changes in immune parameters, but the longitudinal data including the analysis of the PR phase is scarce (15, 20, 21, 23–27), the present study was aimed at analyzing the percentage and absolute counts of innate and adaptive immune cell subsets in peripheral blood and the concentration of different cytokines in plasma over one year after T1D diagnosis, differentiating patients with and without PR to find specific monitoring and predictive biomarkers of this stage. These biomarkers could be of great interest to characterize the immunological mechanisms behind the PR phase and β-cell protection, to monitor the early course of T1D, and to stratify patients with better disease prognosis at the onset of the disease for their selection in clinical trials. Moreover, in the case of non-remitters, these biomarkers would allow for a more personalized therapeutic management to ensure the prevention of early hyperglycemia and to reduce the risk of secondary complications.

## Materials and Methods

### Participants

For the longitudinal study of T1D progression and the characterization of the PR phase, 17 pediatric patients with new-onset T1D (of which 16 completed the study) and 17 age- and sex-matched non-diabetic control subjects were included. For the PR predictive model, 10 additional pediatric patients only at T1D onset (*n* = 27) were selected.

All patients fulfilled the American Diabetes Association classification criteria for T1D (28), with at least one positive anti-islet autoantibody at disease onset [to insulinoma-antigen 2 (IA-2), glutamic acid decarboxylase 65 (GAD65), or zinc transporter 8 (ZnT8)]. Inclusion criteria were 4–18 years of age and normal BMI according to the Spanish BMI pediatric cohort growth chart (29). Exclusion criteria were being under immunosuppressive or anti-inflammatory treatment, type 2 diabetes, pregnancy, compromised kidney function, or liver diseases. Also, candidates were excluded if they presented moderate/severe symptomatic infections or fever in the previous 2–4 weeks before blood withdrawal (i.e., Flu or Covid-19).

### Sample Collection, Longitudinal T1D Follow-Up, and PR

Longitudinal T1D data collection occurred over one year in two University Hospitals. Blood samples of 6 ml were obtained at three different time-points throughout T1D progression of each patient: at disease onset (*n* = 17), at PR (*n* = 11) or 8 months for non-remitter patients (*n* = 6), and at 12 months after disease onset (*n* = 16) in EDTA tubes (BD Biosciences, San Jose, CA, USA) and processed within 6 h. Control samples of 6 ml of blood from non-diabetic subjects (sex and age-related, and with the same exclusion criteria) were acquired simultaneously to the diagnosis of children with T1D. In addition, non-longitudinal blood samples of 3 ml from 10 patients only at T1D onset (6 future remitters, 4 future non-remitters) were obtained for the generation of the PR predictive model and processed as above. At disease onset, all samples (*n* = 27) were collected between 1 and 14 days after diagnosis. After the monthly medical visit, patients were considered to be in PR when they fulfilled the accepted criteria of ≤9 insulin dose-adjusted HbA1c (IDAA1c), an index that is calculated as HbA1c (%) + [4 × insulin dose (U/kg/day)] (30). This phase was identified between 2 and 6 months after diagnosis, and individuals who did not meet the criteria of PR after 8 months, were defined as non-remitters.

### Clinical and Laboratory Testing

Clinical descriptors on each patient and control subject were collected, namely, age, sex, and BMI. BMI data were also expressed as standard deviation score (SDS) for age and sex, based on the Spanish BMI pediatric cohort growth chart data (29). Blood samples from patients with T1D were acquired for centralized measurement of HbA1c, fasting basal and stimulated C-peptide, genetics, and immunology; and insulin requirements were recorded. At the time of T1D diagnosis, HLA typing of DRB1 alleles and islet autoantibodies to IA-2, GAD65, and ZnT8 were determined as previously reported (20). HbA1c was determined by high-performance liquid chromatography (ADAMS A1c HA-8180V, Arkray, MN, USA) in all patients at each time-point. Fasting basal C-peptide was determined by ELISA (Architect i2000, Abbott, IL, USA) in both controls and patients at each time-point. Only at T1D onset, stimulated C-peptide was measured 6 min after i.v. administration of 1 mg glucagon.

### Flow Cytometry

Phenotypic analysis of cellular subpopulations was performed in control subjects and patients with T1D at each time-point. To that end, fresh whole blood samples of 1–2 ml were washed with 15 ml of FACSFlow Sheath Fluid (ThermoFisher Scientific, Waltham, MA, USA), and 100 μl were stained with specific mAbs for 20 min at room temperature and protected from light. The panels of antibodies (**Supplemental Tables 1, 2**) were built as follows: **(1)** T lymphocyte maturation stages panel: CD3-V500, CD4-PerCPCy5.5, CD8-APCH7, CD45RA-FITC, PTK7-PE, CCR7-PECy7, CD31-AF647, CD27-BV421, **(2)** T_REG_ panel: CD45-FITC, CD3-V450, CD4-PerCPCy5.5, CD25-PE, CCR4-PECy7, CD127-AF647, CD45RO-APCH7, HLA-DR-V500, **(3)** T_H_17 lymphocytes panel: CD4-V450, CCR6-PE, CCR7-PECy7, and CCR4-AF647, **(4)** TCR panel: CD3-PerCP, γδ TCR-PE, αβ TCR-FITC, CD8-APCH7, CD4-V450, **(5)** B lymphocyte maturation stages panel: CD3-V450, CD19-V500, CD27-APC, CD21-PE, IgD-FITC, IgM-PerCPCy5.5, **(6)** B lymphocyte subpopulations panel: CD19-PerCPCy5.5, CD24-FITC, CD38-PE, CD27-APC, and **(7)** Innate cells panel: CD45-AF700, CD3-APCH7, CD19-APCH7, CD14-V450, CD16-APC, CD11c-PECy7, CD123-PerCPCy5.5, CD56-PE, HLA-DR-V500, Slan-FITC. After incubation, erythrocytes were lysed for 7 min (Lysing Buffer, BD Biosciences). Samples were then washed and resuspended in FACSFlow Sheath Fluid (ThermoFisher Scientific). Absolute counts (cells/μl) of leukocytes and lymphocytes were obtained using Perfect Count Microspheres of known concentration (Cytognos SL, Salamanca, Spain).

A minimum of 10,000 events per sample and 5,000 beads were acquired using a 3-laser FACS Canto II and a 4-laser LSR Fortessa Flow Cytometers (BD Biosciences) and analyzed using FACSDiva software (BD Biosciences). Necrotic and apoptotic cells were excluded from the analysis based on their FSC-A/SSC-A properties and doublets were excluded by FSC-A/FSC-H. The gating strategy to analyze specific leukocyte subsets was based on international consensus (31). Fluorescence minus one controls were used to define CCR7 expression on T_H_17 lymphocytes and PTK7 expression on recent thymic emigrants. Furthermore, internal reference populations were used as positivity controls in the analysis of CCR7 vs. CD45RA in panel 1, CD27 vs. CD24 in panel 2, CD19 vs. CD21 in panel 3, CCR4 vs. CCR7 in panel 4, and CD45RO vs. CCR4 plus CD45RO vs. HLA-DR in panel 5. Absolute counts were calculated as follows: (%subset/100) × counts of the main subpopulation.

### Serum Cytokine Quantification

Plasma samples were obtained after centrifuging 4–5 ml of whole blood samples twice at 4°C within the first hour after venipuncture (1,900 G for 10 min; 16,000 G for 10 min) and stored at −80°C until use. The BD Cytometric Bead Array (CBA) Human Enhanced Sensitivity Flex Sets for IL-2, IL-6, IL-10, and IL-17A (BD Biosciences) were used to measure cytokine concentration (detection range 274–200,000 fg/ml). Samples were acquired on an LSR Fortessa flow cytometer (BD Biosciences), and data were analyzed using CBA software. The Human TGF-β1 ELISA Kit (FineTest, Wuhan Fine Biotech, Wuhan, China) was used for the quantification of TGF-β1 (detection range 31.25–2,000 pg/ml; sensitivity 18.75 pg/ml).

### Statistical Analysis

Data were tested for normal distribution with the Shapiro–Wilk test and are presented as mean ± SD, mean (min, max), or median and interquartile range, unless stated otherwise. For clinical parameters, a descriptive statistical analysis of the variables was performed. χ² test was used for categorical variables. Differences between two groups were analyzed using the nonparametric 2-tailed Wilcoxon test for paired data and the nonparametric 2-tailed Mann–Whitney test for unpaired data. To test differences among longitudinal data, a mixed effects model was fit with condition as fixed effect, and individual and residual random variation as random effect covariates together with the Tukey’s multiple comparisons test. Kruskal–Wallis test followed by Dunn’s multiple comparisons test was used to compare data between control subjects and each time-point of T1D progression. To establish associations between immunological, clinical, and metabolic variables and the occurrence of PR, simple logistic regressions were developed. Samples with missing data were removed. The OR with the 95% CI was reported as a probability measure. Receiver-operating characteristic (ROC) curves were generated from the logistic regressions to obtain the area under the curve (AUC), which is useful to assess the discrimination ability of the variables. G-test of goodness-of-fit was also employed. To generate a full model containing simultaneously the statistically significant immunological variables, an index was created from the best cut-off values of the ROC curves of each variable. Simple and multiple logistic regressions (adjusted by BMI-SDS) were generated to assess the association between the index and the event of PR. The relationship between covariates was determined with linear regressions. To check the robustness of the results, multicollinearity was examined by the variance inflation factors (VIF), R^2^ with other variables, and Spearman’s correlations between the selected variables. To find statistically significant correlations between different parameters, a two-tailed Spearman’s or Pearson’s test was used. All analyses were performed with GraphPad Prism 9.0 (GraphPad Software Inc, San Diego, CA, USA) and Stata v16.0 (Stata Corporation, College Station, TX, USA). A *P*-value of ≤0.05 was considered statistically significant.

## Results

### Clinical Features and Metabolic Data of Pediatric Patients With T1D

Clinical features and metabolic data from control subjects and patients with T1D at each time-point for the longitudinal study are summarized in **Table 1**. No statistically significant differences were found in age and BMI when compared between control subjects and patients at T1D onset; as expected, they were found in terms of plasma C-peptide concentration (1.3 ± 0.4 vs 0.3 ± 0.2; *P <*0.0001).

**Table 1:** Clinical features and metabolic data of control subjects and patients with T1D included in the longitudinal study.

	Control subjects (*n* = 17)	T1D onset (*n* = 17)	Remitter T1D patients (*n* = 11)		Non-remitter T1D patients (*n* = 6)	
	Baseline	Baseline	PR (2–6 months after baseline)	12 months after baseline	8 months after baseline	12 months after baseline
Age (years)	8.8 ± 3.4	8.7 ± 3.6	9.1 ± 4.3	10.2 ± 4.3**^,xx^	9 ± 2.8	9.2 ± 2.8*
Sex (M/F)	7/10	7/10	5/6	4/6	2/4	2/4
BMI (kg/m^2^)	18.3 ± 4.3	16.8 ± 2.5	17.7 ± 3*	18.6 ± 3.5*	17.2 ± 2.1	17 ± 1.9
BMI-SDS	0.04 (−1.7, 2.7)	−0.5 (−1.6, 0.7)	−0.2 (−1.2, 1.1)*	−0.1 (−1.0, 1.6)	−0.4 (−1.1, 0.3)	−0.5 (−1.0, 0.2)
HbA1c (%)	NA	11.4 ± 2.4	6.9 ± 0.6**	7.1 ± 0.8**/^^^	8.1 ± 0.7*/^xx^	7.9 ± 0.9*/^x^
HbA1c (mmol/mol)	NA	101.3 ± 26.2	51.5 ± 6.4**	54.2 ± 9.1**/^^^	64.5 ± 7.4*/^xx^	63 ± 10.1*/^x^
Insulin dose (U/Kg/day)	NA	0.7 ± 0.2	0.4 ± 0.1**	0.5 ± 0.2^^^^^	0.9 ± 0.1^xxx^	0.85 ± 0.1^xxx^/°°
Basal C-peptide (ng/ml)	1.3 ± 0.4	0.3 ± 0.2^++++^	0.7 ± 0.5^++^	0.3 ± 0.3^++++^	0.3 ± 0.1^+++^	0.15 ± 0.06^+++^/^xx^
Stimulated C-peptide (ng/ml)	NA	0.6 ± 0.4	ND	ND	ND	ND
IDAA1c	NA	14.3 ± 3	8.4 ± 0.5**	9.2 ± 1.3**/^^^^	11.5 ± 1*/^xxx^	11.3 ± 1.2*/^xxx^/°°

Data presented as mean ± SD, or mean (min, max). P-value calculated from Mann–Whitney test when comparing control subjects and patients with T1D (^++^P ≤0.01, ^+++^P ≤0.001 and ^++++^P ≤0.0001), and remitter and non-remitter groups [^x^P ≤0.05, ^xx^P ≤0.01 and ^xxx^P ≤0.001 for comparison with PR; ^P ≤0.05, ^^P ≤0.01 and ^^^P ≤0.001 for comparison with 8 months after baseline (non-remitters); and °°P ≤0.01 for comparison with 12 months after baseline (remitters)]. P-value calculated from Wilcoxon test for comparisons of paired data with disease onset (*P ≤0.05 and **P ≤0.01). BMI, Body Mass Index; F, female; HbA1c, glycated hemoglobin; IDAA1c, insulin dose-adjusted HbA1c; M, male; NA, not applicable; ND, not determined; SDS, standard deviation score.

The 64.7% of patients with T1D presented PR between 2 and 6 months after the diagnosis, with an IDAA1c value equal to or less than 9, while the rest did not experience it after 12 months of follow-up. During the PR phase, and in comparison to disease onset, patients showed increased BMI and BMI-SDS values, and a non-significant increase in basal C-peptide concentration (0.7 ± 0.5 vs 0.3 ± 0.2; ns), although the levels were still much lower than those of the control group (0.7 ± 0.5 vs 1.3 ± 0.4; *P <*0.01). After a year follow-up, 50% of these remitter patients were still in remission. There were no significant differences in terms of age, sex, and BMI between patients with and without PR at 8 months of follow-up. Unlike patients in remission, non-remitter patients (IDAA1c >9) did not present an increase in basal C-peptide concentration after diagnosis (0.3 ± 0.1 vs 0.3 ± 0.2; ns), indicating some β-cell recovery only in remitter patients.

After 12 months of follow-up, the group of remitter patients showed lower levels of basal C-peptide than levels during remission; however, these doubled those of the non-remitters (0.3 ± 0.3 vs 0.15 ± 0.06; ns). Moreover, the group of remitter patients still needed a lower daily dose of total insulin than the group of non-remitter patients (0.5 ± 0.2 vs 0.85 ± 0.1; *P <*0.01) and maintained the increased BMI values regarding the disease onset (18.6 ± 3.5 vs 16.8 ± 2.5; *P* ≤0.05). At 12 months, both remitters and non-remitters maintained practically equal the HbA1c values reached during the PR phase or at 8 months from diagnosis, respectively.

Clinical features and metabolic data from patients with T1D at disease onset for the PR predictive study are summarized in **Table 2**. Differences at T1D onset between remitters and non-remitters were found in terms of insulin dose (0.6 ± 0.2 vs 0.8 ± 0.1, *P <*0.01) and stimulated C-peptide (0.9 ± 0.8 vs 0.5 ± 0.5, *P* ≤0.05).

**Table 2:** Clinical features and metabolic data of patients with T1D included in the PR predictive model study at disease onset separated by future remitters and non-remitters.

	T1D onset (future PR) (*n* = 17)	T1D onset (future non-PR) (*n* = 10)	*P*-value
Age (years)	9.8 ± 5	6.4 ± 3.4	0.07
Sex (M/F)	8/9	4/6	0.72
BMI (kg/m^2^)	17.7 ± 3.0	15.7 ± 1.7	0.12
BMI-SDS	−0.5 (−1.8, 0.7)	−0.7 (−1.7, 0.5)	0.54
HbA1c (%)	11.1 ± 2.5	11.4 ± 1.7	0.61
HbA1c (mmol/mol)	98.1 ± 27.5	100.6 ± 18.6	0.61
Insulin dose (U/Kg/day)	0.6 ± 0.2	0.8 ± 0.1	0.01**
Basal C-peptide (ng/ml)	0.4 ± 0.3	0.3 ± 0.2	0.15
Stimulated C-peptide (ng/ml)	0.9 ± 0.8	0.5 ± 0.5	0.03*
IDAA1c	14.5 ± 3.2	14.7 ± 2.0	0.98

Data presented as mean ± SD, or mean (min, max). *P*-value calculated from Mann–Whitney test when comparing baseline time-points (T1D onset) between remitters and non-remitters (*P ≤0.05 and **P ≤0.01). BMI, Body Mass Index; F, female; HbA1c, glycated hemoglobin; IDAA1c, insulin dose-adjusted HbA1c; M, male; SDS, standard deviation score.

### Increase in Effector Memory CD4^+^ and CD8^+^ T Lymphocytes at the PR Phase

**Figure 1:**
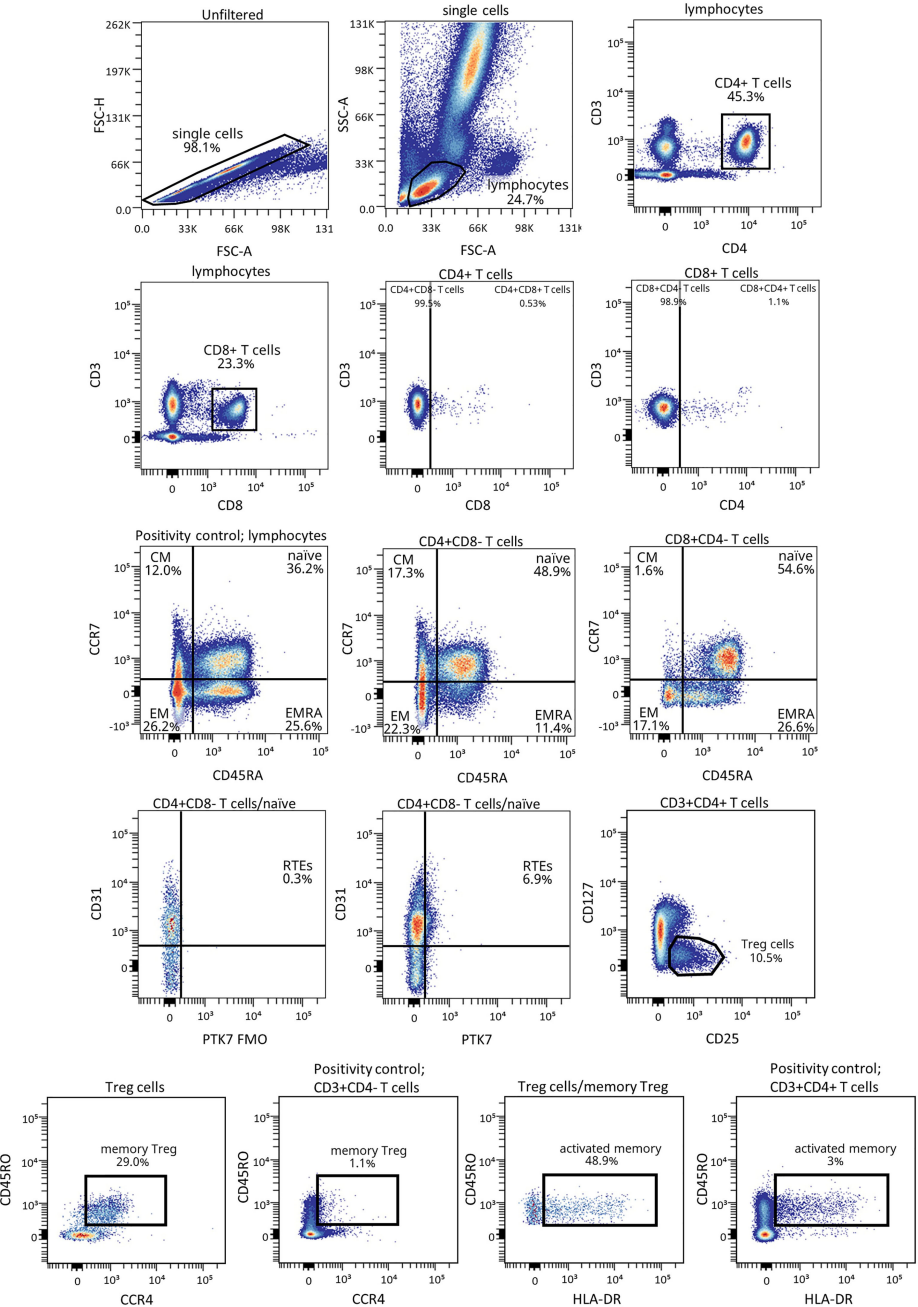
Representative gating strategy for the T lymphocyte maturation stages and T_REG_ panels. Gating strategy used to analyze the percentages of the different CD4^+^ and CD8^+^ T cell subsets based on the expression of the markers CD3, CD4, CD8, CD45RA and CCR7, and of CD127, CD25, CD45RO, CCR4 and HLA-DR for T_REG_ subsets within CD4^+^ T cells. CD31 and PTK7 were used to characterize RTEs. Positivity controls for CCR7 vs CD45RA using lymphocytes, for CD45RO vs CCR4 using CD4^-^ T cells and for CD45RO vs HLA-DR using CD4^+^ T cells as internal reference populations. FMO control for PTK7 was used.

**Figure 2:**
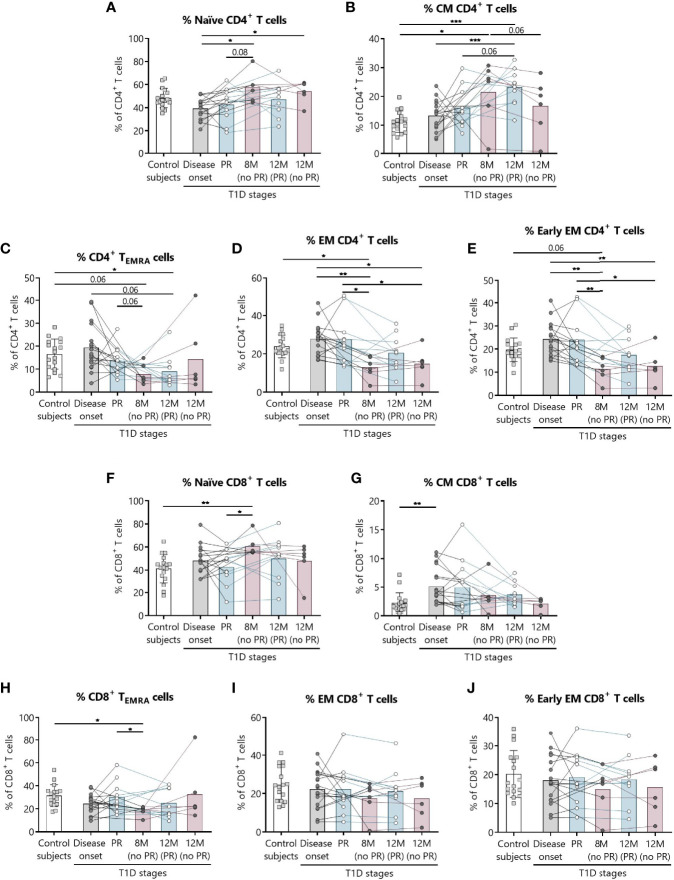
Percentages of CD4^+^ and CD8^+^ T lymphocyte subsets are altered at the initial stages of T1D. For CD4^+^ and CD8^+^ T lymphocytes, respectively, the percentages (%) of **(A, F)** naïve T lymphocytes, **(B, G)** CM T lymphocytes, **(C, H)** T_EMRA_ lymphocytes, **(D, I)** EM T lymphocytes, and **(E, J)** early EM T lymphocytes were determined in peripheral blood of control subjects and patients with T1D at different time-points. Squares represent controls (*n* = 17) (white bar), and patients are represented by light gray dots at disease onset (*n* = 17) (gray bar), white dots during PR (*n* = 11) and for remitter patients at 12 months (12 M PR) (*n* = 10) (blue bars), and dark gray dots for non-remitter patients at 8 months (8 M no PR) (*n* = 6) and 12 months (12 M no PR) (*n* = 6) (pink bars). Bar graphs show mean percentage values. Each symbol represents an individual patient. Lines link the same patient throughout the time-points. *P ≤ 0.05, **P < 0.01, ***P < 0.001 after mixed effects model with Tukey’s post-hoc test for longitudinal data, Kruskal–Wallis with Dunn’s post-hoc test for comparisons between control subjects and the different T1D time-points, or 2-tailed Mann–Whitney test for comparisons between two unpaired groups of data. *P* ≤0.05 is considered significant.

**Figure 1** shows the gating strategy followed for the antibody panels of T lymphocyte maturation stages and T_REG_. The analysis of the maturation stages of both CD4^+^ and CD8^+^ T lymphocytes (naïve, central memory (CM), effector memory (EM), and terminally differentiated effector memory T (T_EMRA_) lymphocytes), revealed statistically significant changes in blood from patients during the PR phase (**Figure 2**).

Regarding CD4^+^ T lymphocytes, non-remitter patients presented higher percentages of naïve CD4^+^ T lymphocytes both at 8 and 12 months in comparison to patients at disease onset (*P* ≤0.05) (**Figure 2A**). While the percentage of CM CD4^+^ T lymphocytes tended to increase with disease progression for the remitter ones—especially at 12 months after T1D onset in comparison to the diagnosis and the control group (*P <*0.001)—the percentage of this subpopulation tended to decrease at 12 months for non-remitter patients in comparison to the time-point of 8 months, where levels were also higher than those of the control group (*P* ≤0.05). However, no significant differences were observed between remitters and non-remitters at 8 months (**Figure 2B**).

The main findings observed during the PR phase when compared with non-remitter patients were higher percentages of EM and early EM CD4^+^ T lymphocytes and CD8^+^ T_EMRA_ lymphocytes (**Figures 2D, E, H**). The percentage of CD4^+^ T_EMRA_ lymphocytes decreased at 12 months for remitter patients in comparison to controls (*P* ≤0.05), but tended to be higher during the PR phase in comparison to non-remitters at 8 months (**Figure 2C**). Peripheral EM and early EM CD4^+^ T lymphocytes behaved similarly (**Figures 2D, E**). The percentages of both subpopulations were significantly increased during the PR phase in comparison to 8 months (*P* ≤0.05 and *P <*0.01, respectively) and 12 months (*P* ≤0.05) for non-remitter patients. The latter ones at 8 months also showed decreased percentages in comparison to disease onset (*P <*0.01) and control subjects (*P* ≤0.05 and *P* = 0.06, respectively), and at 12 months than patients at diagnosis (*P* ≤0.05 and *P <*0.01, respectively).

Concerning the absolute counts, only CD4^+^ T_EMRA_ lymphocytes significantly increased during the PR phase in comparison to 8 months for non-remitters. No other significant difference was observed between patients with and without PR (**Supplemental Figures 1A–E**).

Regarding CD8^+^ T lymphocytes, patients at the PR phase presented lower percentages of naïve CD8^+^ T lymphocytes compared to patients without PR at 8 months (*P* ≤0.05). Moreover, non-remitter patients showed a higher percentage of this subpopulation than controls (*P <*0.01) (**Figure 2F**). Contrary to what was observed in the CM CD4^+^ T lymphocyte subpopulation, the percentage of CM CD8^+^ T lymphocytes tended to decrease with T1D progression and showed a significant increase at disease onset compared to the control group (*P <*0.01) (**Figure 2G**). As mentioned before, the percentage of CD8^+^ T_EMRA_ lymphocytes was lower at 8 months for non-remitter patients in comparison to patients at the PR phase (*P* ≤0.05) and controls (*P* ≤0.05) (**Figure 2H**). Lastly, and regarding the percentage of EM and early EM CD8^+^ T lymphocytes, no differences were found between groups (**Figures 2I, J**).

Concerning the absolute counts of these subsets, no significant differences were found between patients with and without PR (**Supplemental Figures 1F–J**).

As for total CD4^+^ T lymphocytes, their percentage increased at 8 months for non-remitter patients when compared to patients during the PR phase (*P <*0.01), to remitter patients at 12 months of follow-up (*P* ≤0.05) and controls (*P* ≤0.05), but no differences between remitters and non-remitters were found regarding their absolute numbers (**Supplemental Figures 2A, B**). Concerning the absolute counts and percentages of total CD8^+^ T lymphocytes, late EM CD4^+^ and CD8^+^ T lymphocytes, recent thymic emigrants, γδ TCR cells, and T_H_17 lymphocytes, no significant differences were found between patients with and without PR (**Supplemental Figures 2C–N**). Gating strategies for the antibody panels of TCR and T_H_17 lymphocytes are depicted in **Supplemental Figures 3, 4**.

### Decrease in Total and Memory T_REG_ at the PR Phase

Since T_REG_ are expected to be implicated in the immunoregulatory mechanism behind the PR phase (32), we analyzed peripheral T_REG_ subsets, namely, total, memory, and activated T_REG_. Furthermore, representative confirmatory staining for FoxP3 within the T_REG_ population can be found in **Supplemental Figure 5**, where 74.6% of these cells do express it.

**Figure 3:**
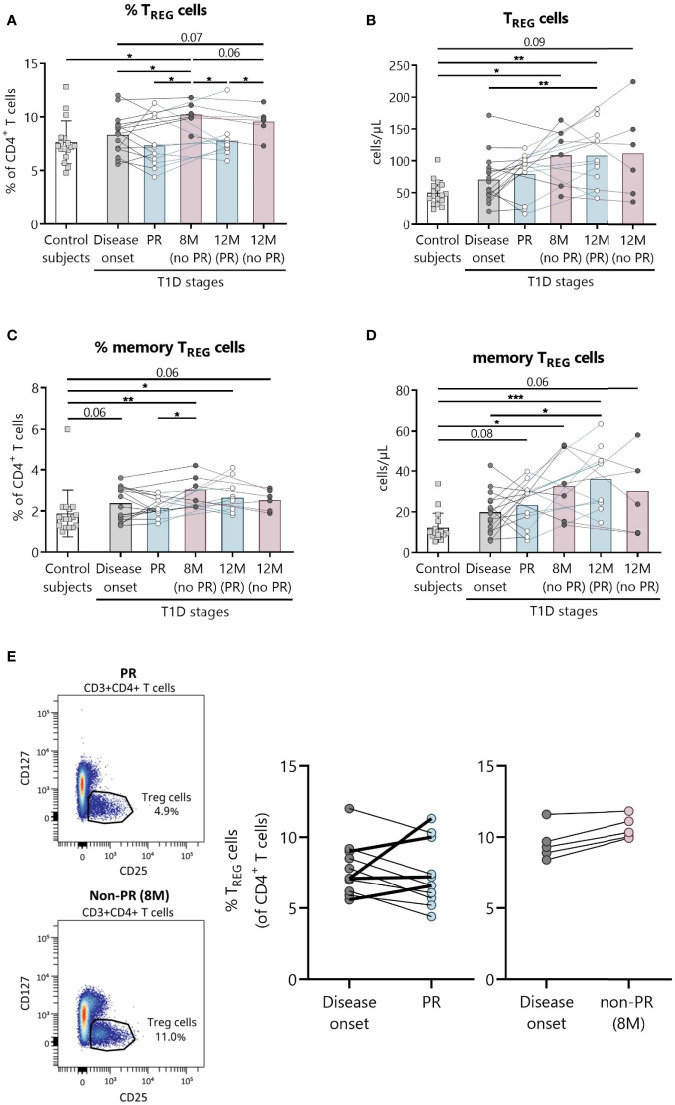
Peripheral blood T_REG_ are quantitatively altered at the initial stages of T1D. Percentages (%) and concentrations (cells/µl) of **(A, B)** T_REG_ and of **(C, D)** memory T_REG_ were determined in peripheral blood of controls and patients with T1D at different time-points. Squares represent controls (*n* = 17) (white bar), and patients are represented by light gray dots at disease onset (*n* = 16) (gray bar), white dots during PR (*n* = 11) and for remitter patients at 12 months (12 M PR) (*n* = 10) (blue bars), and dark gray dots for non-remitter patients at 8 months (8 M no PR) (*n* = 6) and 12 months (12 M no PR) (*n* = 6) (pink bars). **(E)** Representative plots for the difference in the T_REG_ percentage between PR and non-PR (8 M), and changes in this cell population from the disease onset to the PR phase or non-PR (8 M). Bar graphs show mean percentage or absolute count values. Each symbol represents an individual patient. Lines link the same patient throughout the time-points. *P ≤0.05, **P < 0.01, ***P < 0.001 after mixed effects model with Tukey’s post-hoc test for longitudinal data, Kruskal–Wallis with Dunn’s post-hoc test for comparisons between control subjects and the different T1D time-points, or 2-tailed Mann–Whitney test for comparisons between two unpaired groups of data. *P* ≤0.05 is considered significant.

The main findings observed in patients with T1D at the PR phase when compared with patients without the PR were lower percentages of both total and memory T_REG_ (**Figures 3A, C**). Specifically, the percentage of total T_REG_ increased at 8 months from diagnosis for patients without PR in comparison with both the control group (*P* ≤0.05) and patients at disease onset (*P* ≤0.05). Furthermore, levels at this time-point are higher than patients in PR, both during that phase (*P* ≤0.05) and 12 months after diagnosis (*P* ≤0.05). After a year follow-up, patients who did not present PR continued to show a higher percentage of total T_REG_ than patients who presented remission (*P* ≤0.05) (**Figure 3A**). Regarding their absolute numbers, total T_REG_ increased with the disease condition in comparison to controls, finding statistically significant differences at 8 months for patients without PR (*P* ≤0.05), and at 12 months for patients that experienced the PR phase (*P <*0.01), whose levels were also higher than those of patients at disease onset (*P <*0.01) (**Figure 3B**).

Concerning memory T_REG_, they increased in percentage compared to the control group at 8 months for patients without remission (*P <*0.01), and at 12 months for patients who experienced PR (*P* ≤0.05). As mentioned above, patients without remission at 8 months present a higher percentage of memory T_REG_ than patients during PR (*P* ≤0.05) (**Figure 3C**). Their absolute numbers behaved similarly, increasing with the disease condition both at 12 months after diagnosis for remitter patients (*P <*0.001) and at 8 months for non-remitters (*P* ≤0.05) when compared to controls. Also, their numbers were higher at 12 months for remitter patients than at T1D onset (*P* ≤0.05) (**Figure 3D**).

As for activated T_REG_, no differences were found between patients with and without PR (**Supplemental Figure 6**).

A different tendency on the percentage of T_REG_ from disease onset (baseline) to PR was observed for 4 children, who increased this cell subpopulation in contrast to the other remitter patients, and as non-remitter patients did (**Figure 3E**). Since those 4 children in PR present the same behavior as the non-remitters at 8 months, differences between the two groups of remitter patients were investigated. Interestingly, these 4 children with increasing percentages of T_REG_ from baseline were all males, and they were the youngest (mean 5.5 vs 11.1 years, *P* ≤0.05). Moreover, they presented lower basal C-peptide concentrations (mean 0.28 vs 1.03, *P* ≤0.05) and BMI values (mean 15.6 vs 18.97, *P* ≤0.05), required lower doses of insulin (mean 0.28 vs 0.44, *P* ≤0.05), and presented a tendency of a higher percentage of HbA1c in comparison to the other 7 remitters with decreasing percentages of T_REG_ from baseline (**Supplemental Table 3**).

### Increase in Regulatory B Lymphocyte Subpopulations After One-Year Follow-Up for Patients That Experienced the PR Phase

**Figure 4:**
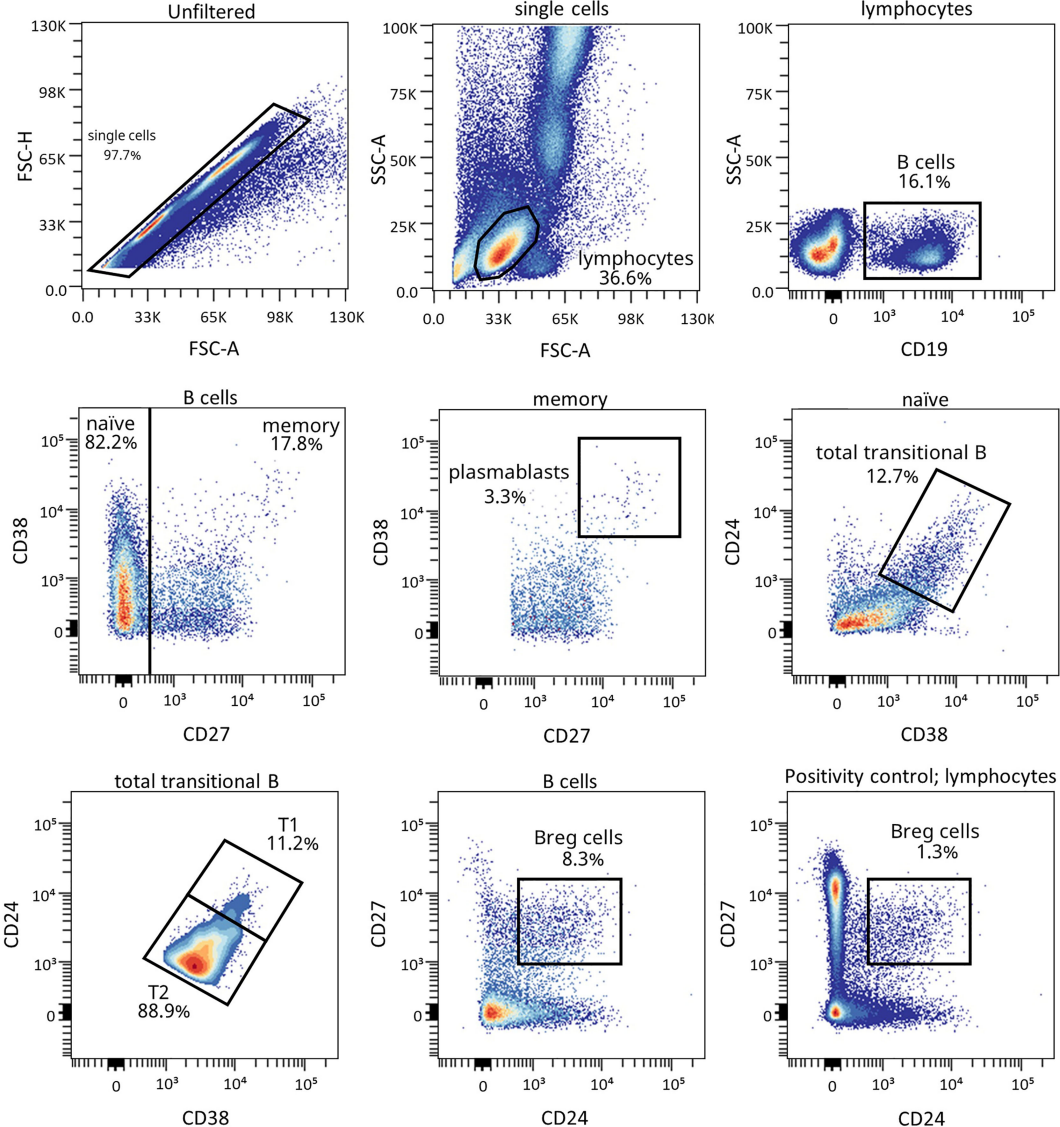
Representative gating strategy for the B cell subpopulations panel. Gating strategy used to analyze the percentages of transitional B cells (total, T1, and T2), B_REG_ and plasmablasts based on the expression of the markers CD19, CD27, CD38, and CD24. Positivity control for CD27 vs CD24 using lymphocytes as an internal reference population.

**Figure 5:**
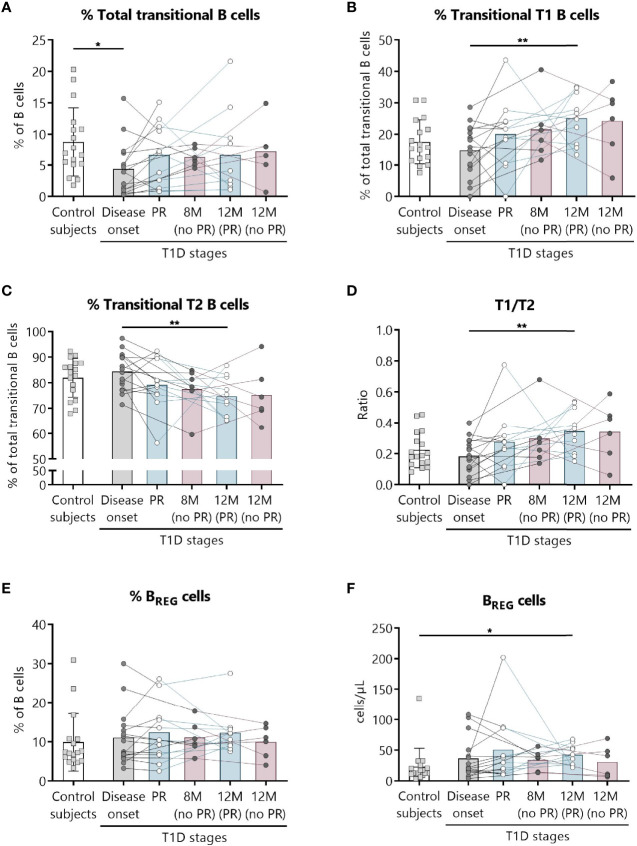
Two regulatory subsets of B lymphocytes are quantitatively altered at the initial stages of T1D. Percentages (%) of **(A)** total transitional B lymphocytes and of their subdivision into **(B)** T1, and **(C)** T2, **(D)** the T1/T2 ratio, and **(E, F)** the percentage (%) and concentration (cells/µl) of B_REG_ were determined in peripheral blood of controls and patients with T1D at different time-points. Squares represent controls (*n* = 17) (white bar), and patients are represented by light gray dots at disease onset (*n* = 16) (gray bar), white dots during PR phase (*n* = 11) and for remitter patients at 12 months (12 M PR) (*n* = 10) (blue bars), and dark gray dots for non-remitter patients at 8 months (8 M no PR) (*n* = 6) and 12 months (12 M no PR) (*n* = 6) (pink bars). Bar graphs show mean percentage or absolute count values. Each symbol represents an individual patient. Lines link the same patient throughout the time-points. *P ≤ 0.05, **P < 0.01 after mixed effects model with Tukey’s post-hoc test for longitudinal data, or Kruskal–Wallis with Dunn’s post-hoc test for comparisons between control subjects and the different T1D time-points. *P* ≤0.05 is considered significant.

Because of the described role for B lymphocytes in T1D pathogenesis, we next examined different naïve and memory B lymphocyte subsets. **Figure 4** shows the gating strategy followed for the antibody panel of B lymphocyte subpopulations. Interestingly, two B lymphocyte subsets with regulatory functions—transitional T1 B lymphocytes and regulatory B cells (B_REG_)—were increased at 12 months for remitter patients (**Figure 5**). The percentage of total transitional B lymphocytes was substantially decreased at disease onset when compared to the control group (*P* ≤0.05), and their levels tended to recover with time (**Figure 5A**). Within this subpopulation, transitional T1 B lymphocytes increased in percentage at 12 months for patients that experienced the PR phase when compared to the diagnosis (*P <*0.01), while transitional T2 B lymphocytes decreased (*P <*0.01) (**Figures 5B, C**). Moreover, at this time-point, patients presented a higher T1/T2 ratio than patients at T1D onset (*P <*0.01) (**Figure 5D**).

The gating strategy followed for the antibody panel of B lymphocyte maturation stages is depicted in **Supplemental Figure 7**. Concerning the percentages and absolute counts of total B lymphocytes, naïve B lymphocytes, CD21^−/low^ naïve B lymphocytes, mature naïve B lymphocytes, memory B lymphocytes—namely, exhausted, unswitched, switched, and IgM memory B lymphocytes—(**Supplemental Figures 8, 9**) and plasmablasts (**Supplemental Figure 10**), no differences were found between patients with and without PR.

### Innate Immune Cells Are Altered at the Initial Stages of T1D and During the PR Phase

**Figure 6:**
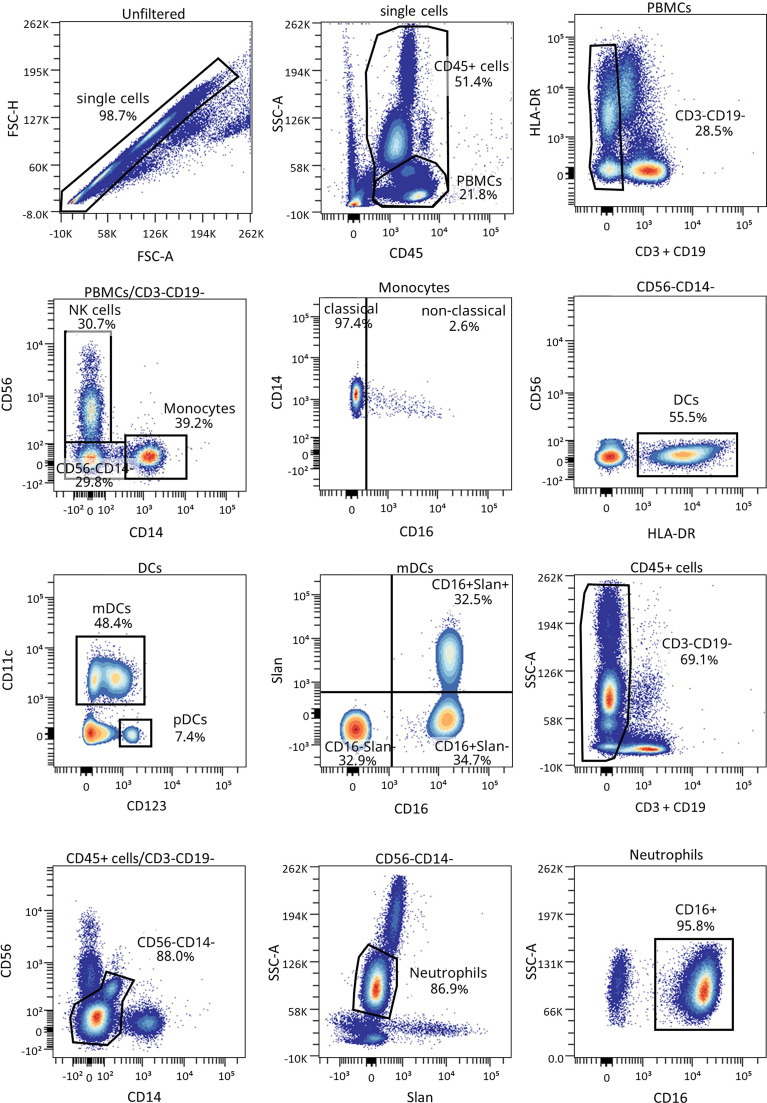
Representative gating strategy for the innate cells panel. Gating strategy used to analyze the percentages of DCs, monocytes, and neutrophils. Cells were first gated for singlets, PBMCs, and CD3^-^CD19^-^ cells. With the use of CD56 and CD14, cells were divided into NK cells and monocytes, which were both further divided into their subsets with the use of CD16, and DCs (lineage negative and HLA-DR^+^). Different subsets of DCs (mDC and pDC) were analyzed using CD123 and CD11c, and mDCs were further divided into their subsets with the use of CD16 and Slan.

**Figure 7:**
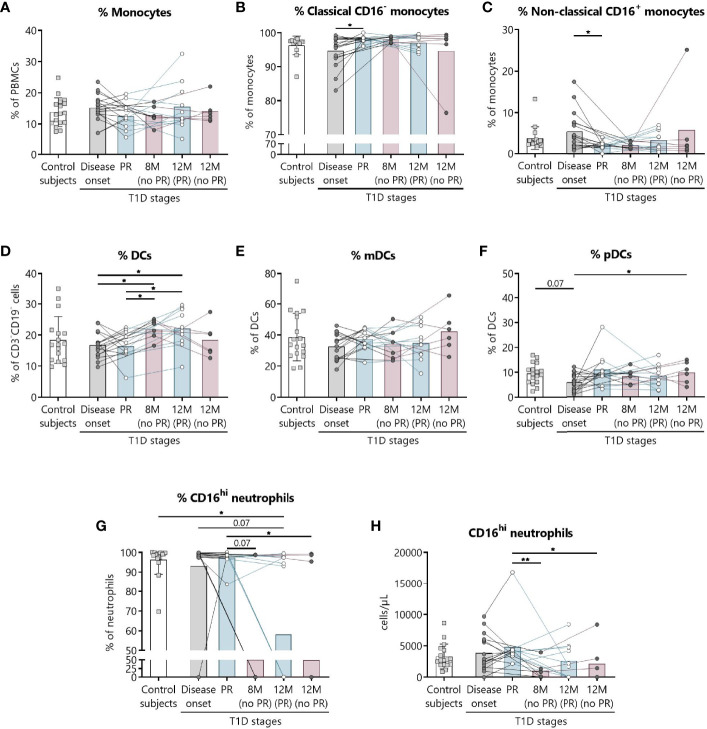
Innate cells are quantitatively altered at the initial stages of T1D. Percentages (%) of **(A)** monocytes and their subsets **(B)** classical, and **(C)** non-classical, of **(D)** DCs and their subsets **(E)** mDCs, and **(F)** pDCs, and **(G, H)** the percentage (%) and concentration (cells/µl) of CD16^hi^ neutrophils were determined in peripheral blood of control subjects and patients with T1D at different time-points. Squares represent controls (*n* ≥16) (white bar), and patients are represented by light gray dots at disease onset (*n* = 17) (gray bar), white dots during PR (*n* = 11) and for remitter patients at 12 months (12 M PR) (*n* = 10) (blue bars), and dark gray dots for non-remitter patients at 8 months (8 M no PR) (*n* = 6) and 12 months (12 M no PR) (*n* = 6) (pink bars). Bar graphs show mean percentage or absolute count values. Each symbol represents an individual patient. Lines link the same patient throughout the time-points. *P ≤ 0.05, **P < 0.01 after mixed effects model with Tukey’s post-hoc test for longitudinal data, Kruskal–Wallis with Dunn’s post-hoc test for comparisons between control subjects and the different T1D time-points, or 2-tailed Mann–Whitney test for comparisons between two unpaired groups of data. *P* ≤ 0.05 is considered significant.

We have previously shown the potential that subsets of NK cells have as biomarkers of T1D progression and PR (20). Thus, changes in neutrophils and different subsets of monocytes and DCs were investigated. **Figure 6** shows the gating strategy followed for the antibody panel of innate cells. Although no significant differences were found between groups regarding the percentage of total monocytes (**Figure 7A**), we found an increase in the percentage of classical CD16^−^ monocytes during the PR phase in comparison to disease onset (*P* ≤0.05), and a subsequent decrease in the percentage of non-classical CD16^+^ monocytes (*P* ≤0.05) (**Figures 7B, C**).

The percentage of total DCs increased after 8 months from diagnosis for non-remitter patients in comparison to patients at PR (*P* ≤0.05) and to patients at disease onset (*P* ≤0.05). Those significant increases can be also observed 12 months after diagnosis for remitter patients (**Figure 7D**). When analyzing the two main subsets of DCs, the myeloid (mDCs) and the plasmacytoid (pDCs) ones, no significant differences were found between groups regarding mDCs (**Figure 7E**), but pDCs increased at 12 months for non-remitter patients when compared to diagnosis (*P* ≤0.05) (**Figure 7F**).

Concerning the absolute numbers of monocytes and their subsets and mDCs and pDCs, no differences were found between patients with and without PR (**Supplemental Figures 11A–C, E, F**). However, one year after onset, non-remitters presented lower counts of total DCs than remitters (*P* ≤0.05) (**Supplemental Figure 11D**). When further subdividing the mDC subset according to the expression of Slan and CD16, no differences were found between patients with and without PR (**Supplemental Figures 12A–F**).

Finally, we detected that the percentage and counts of CD16^hi^ neutrophils remained constant between the control group, patients at disease onset, and patients at PR. Interestingly, the percentage and absolute numbers of CD16^+^ neutrophils decreased in 3 out of 6 non-remitter patients both at 8 months and 12 months from diagnosis in comparison to patients at the PR phase (for percentage, *P* = 0.07 and *P* ≤0.05, respectively; for absolute numbers, *P <*0.01 and *P* ≤0.05, respectively). For 4 out of 10 remitter patients after a year of follow-up, there was also a decrease in the percentage of CD16^hi^ neutrophils when compared to controls (*P* ≤0.05) (**Figures 7G, H**).

### Increase in the Concentration of Cytokines at T1D Onset

**Figure 8:**
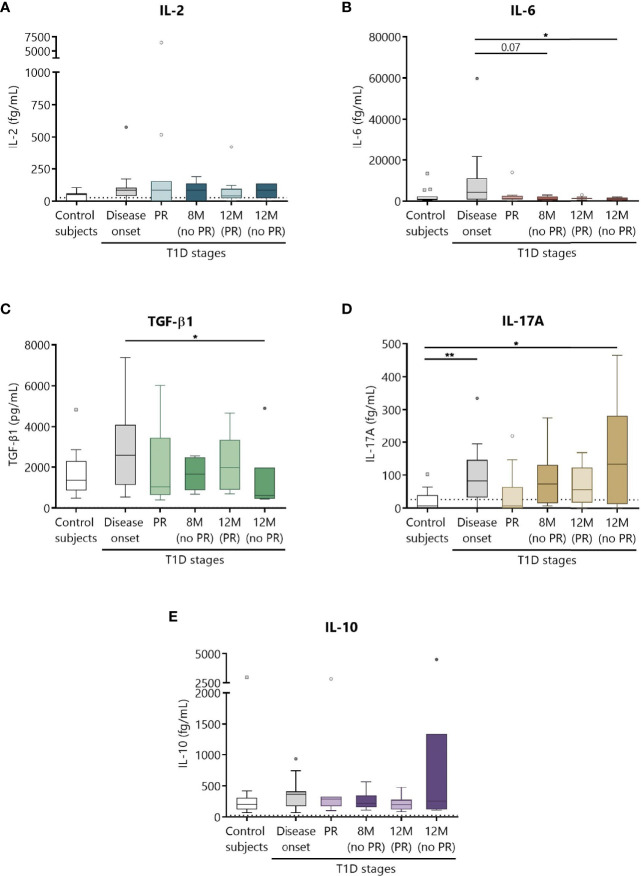
Circulating cytokine concentrations at different stages of T1D. Plasma from control subjects and patients with T1D at different time-points was obtained to quantitatively determine the concentrations of **(A)** IL-2, **(B)** IL-6, **(D)** IL-17A, and **(E)** IL-10 by CBA and of **(C)** TGF-β1 by ELISA. Uncolored boxes represent controls (*n* = 17), and patients are represented by gray boxes at disease onset (*n* = 17), light-colored boxes at PR (*n* = 11) and for remitter patients at 12 months (12 M PR) (*n* = 10), and dark-colored boxes for non-remitter patients at 8 months (8 M no PR) (*n* = 6) and 12 months (12 M no PR) (*n* = 6). Data are presented as box-and-whisker plots. Boxes indicate the first and third quartiles. The horizontal bar in the box indicates the median. Whiskers are drawn using Tukey’s criteria of 1.5× the interquartile range. Outliers beyond the whiskers are shown. *P ≤ 0.05, **P <0.01 after mixed effects model with Tukey’s post-hoc test for longitudinal data, or Kruskal–Wallis with Dunn’s post-hoc test for comparisons between control subjects and the different T1D time-points. *P* ≤ 0.05 is considered significant.

Different cytokines involved in T1D, namely, IL-2, IL-6, TGF-β1, IL-17A, and IL-10 were prospectively analyzed in the plasma of patients as potential biomarkers of PR (**Figure 8**). Although no statistically significant differences were found between patients with and without PR, a tendency for higher levels of all cytokines at T1D onset compared to controls was observed. No differences were noticed regarding the concentration of IL-2 between the different groups (**Figure 8A**). Concerning IL-6, patients at disease onset presented a significant increase in comparison to non-remitters at 12 months (*P* ≤0.05) (**Figure 8B**). Moreover, the higher concentration of TGF-β1 found at diagnosis tended to decrease with disease progression, especially during the PR phase and at 12 months for patients without PR (*P* ≤0.05) (**Figure 8C**). Regarding IL-17A, a statistically significant increase at T1D onset was found when compared to controls (*P <*0.01). Interestingly, remitter patients tended to maintain lower levels of this cytokine in comparison to non-remitter patients, even at a time-point of 12 months. After a year follow-up, non-remitter patients also showed higher IL-17A levels than the control group (*P* ≤0.05) (**Figure 8D**). Finally, IL-10 remained constant throughout the different groups (**Figure 8E**).

### Percentages of T_REG_, DCs, and Monocytes, and the Total Daily Insulin Dose at T1D Onset Could Serve as Predictive Biomarkers of PR

**Figure 9:**
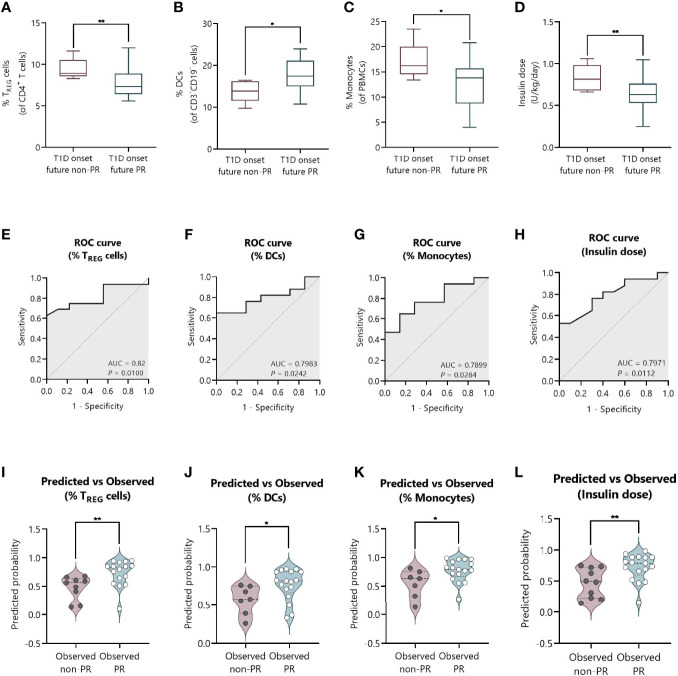
The percentage of T_REG_, DCs, and monocytes, and the total daily insulin dose at T1D onset can discriminate between future remitters and non-remitters. **(A–D)** Box-and-whisker plots showing the percentage levels of **(A)** T_REG_, **(B)** DCs, and **(C)** monocytes, and **(D)** insulin dose at T1D onset separated by future remitters (blue boxes) and non-remitters (pink boxes). Boxes indicate the first and third quartiles and whiskers range from minimum to maximum values. The horizontal bar in the box indicates the median. **(E–H)** ROC curves plotting sensitivity and 1-specificity for detecting children with PR using percentages of **(E)** T_REG_, **(F)** DCs, and **(G)** monocytes, and **(H)** insulin dose. The AUC is indicated, being a measure of how well a quantitative test can distinguish between patients with and without PR. **(I–L)** Violin plots showing the frequency distribution of predicted probabilities for both the observed PR and non-PR groups regarding the percentage of **(I)** T_REG_, **(J)** DCs, and **(K)** monocytes, and **(L)** insulin dose. Each dot represents an individual patient. Violin plots show the median (thick dashed line) and first and third quartiles (thin dashed lines). *P ≤ 0.05, **P < 0.01 after 2-tailed Mann–Whitney test. *P* ≤ 0.05 is considered significant. *N* = 24–27.

We next tested the predictive capacity of the PR phase of all the immune cell subsets, cytokines, and metabolic and clinical parameters analyzed at T1D onset. First, simple logistic regression analyses were used to determine the association between the event of PR and each of the parameters (**Table 3**). While none of the different cytokine concentrations in plasma could predict the PR event, the percentage of T_REG_ (OR = 0.45, 95% CI: 0.17 to 0.83, *P* ≤0.05), monocytes (OR = 0.71, 95% CI: 0.46 to 0.94, *P* ≤0.05), and DCs (OR = 1.39, 95% CI: 1.05 to 2.07, *P* ≤0.05) did, meaning that having increasing percentages of T_REG_ and monocytes would correspond with lower odds of being in remission. Contrarily, the likelihood of being in PR is 39% higher for each increase in the percentile of DCs. As for the metabolic parameters, only the insulin dose could predict the event of PR (OR = 7.3e^−4^, 95% CI: 4.18e^−007^ to 0.16, *P* ≤0.05), meaning that the requirement of higher doses of exogenous insulin would correspond with lower odds of being in remission. In fact, these cell percentages and insulin dose values at T1D onset, separated by remitters and non-remitters, showed how those remitter patients were diagnosed with lower percentages of T_REG_ (*P <*0.01) and monocytes (*P* ≤0.05), a higher percentage of DCs (*P* ≤0.05), and lower doses of exogenous insulin (*P <*0.01) compared to those non-remitter patients (**Figures 9A–D**). Moreover, the G-test was 6.982 for T_REG_ (*P <*0.01), 6.152 for monocytes (*P* ≤0.05), 5.554 for DCs (*P* ≤0.05), and 7.501 for the insulin dose (*P <*0.01), having those parameters the highest G-test scores of all the analyzed parameters (**Table 3**). Then, the relationship between covariates (age, sex, BMI, HbA1c, basal and stimulated C-peptide, IDAA1c, insulin dose, and immunological variables) was determined with linear regressions, finding that none of the parameters were predictive of each other, except for insulin dose and T_REG_ percentage (**Supplemental Table 4**). Although the results were not significant, age and BMI at T1D onset could also serve as potential predictors of PR, with a 20% increase in the odds of being in PR for each year added (*P* = 0.08) and with a 42% increase in the odds for each BMI unit increase (*P* = 0.08). No other metabolic parameter or cell subpopulation was able to predict the event of PR (**Table 3**, **Supplemental Table 5**).

**Table 3:** Simple logistic regressions for determinants of PR at T1D onset.

Variable	Coefficient	SE	OR [95% CI]	|Z|	*P*-value^G^	G test	*P*-value^H^
**Immunological variables**							
** Peripheral immune cell subsets (%)**							
T_REG_^A^	−0.798	0.377	0.45 [0.17 to 0.83]	2.116	0.03*	6.982	0.01*
Monocytes^B^	−0.332	0.172	0.71 [0.46 to 0.94]	1.936	0.05*	6.152	0.01*
DCs^B^	0.330	0.168	1.39 [1.05 to 2.07]	1.959	0.05*	5.554	0.02*
** Cytokine concentration (in plasma)^C^**							
IL-17A (fg/ml)	0.009	0.008	1.00 [0.99 to 1.02]	1.153	0.25	1.696	0.19
IL-10 (fg/ml)	0.0004	0.002	1.00 [0.99 to 1.00]	0.169	0.87	0.029	0.86
IL-2 (fg/ml)	0.001	0.004	1.00 [0.99 to 1.01]	0.285	0.78	0.088	0.77
IL-6 (fg/ml)	4.823e^−005^	6.575e^−005^	1.00 [1.00 to 1.00]	0.733	0.46	0.888	0.35
TGF-β (ng/ml)	−4.324e^−005^	0.0002	1.00 [0.99 to 1.00]	0.180	0.86	0.032	0.86
**Clinical and metabolic variables**							
Age (years)	0.189	0.108	1.20 [0.99 to 1.55]	1.747	0.08	3.779	0.05*
Sex^D^	0.288	0.808	1.33 [0.28 to 6.90]	0.36	0.72	0.128	0.72
BMI (kg/m^2^)	0.352	0.204	1.42 [1.00 to 2.28]	1.726	0.08	3.928	0.05*
BMI-SDS	0.492	0.638	1.64 [0.48 to 6.26]	0.771	0.441	0.610	0.44
HbA1c (%)	−0.049	0.183	0.95 [0.65 to 1.37]	0.265	0.79	0.070	0.79
C-peptide (ng/ml)	2.436	1.983	11.43 [0.41 to 1170]	1.228	0.22	1.926	0.17
Stimulated C-peptide (ng/ml)^B^	2.191	1.427	8.94 [0.96 to 248.8]	1.535	0.12	3.687	0.05*
IDAA1c	−0.031	0.149	0.96 [0.71 to 1.30]	0.21	0.83	0.044	0.83
Insulin dose (U/kg/day)	−7.217	3.174	7.3e^−4^ [4.18e^−007^ to 0.16]	2.274	0.02*	7.501	0.01*
α-IA-2 autoAb^E,F^	0.470	1.524	1.60 [0.08 to 31.77]	0.31	0.756	0.09	0.76
α-GAD65 autoAb^E,F^	0.470	1.524	1.60 [0.08 to 31.77]	0.31	0.76	0.09	0.76
α-ZnT8 autoAb^C,F^	−0.133	1.069	0.87 [0.10 to 7.11]	−0.12	0.90	0.02	0.90

^A^n = 25; ^B^n = 24; ^C^n = 17; ^D^Ref.: female; ^E^n = 15; ^F^Categorical variable; ^G^P-value from OR; ^H^P-value from G test. BMI, Body Mass Index; HbA1c, glycated hemoglobin; IDAA1c, insulin dose-adjusted HbA1c; SDS, standard deviation score; SE, standard error. *P ≤0.05.

**Figures 9E–H** show the ROC curves depicting the sensitivity by 1-specificity and the AUC values for the distinction of the PR phase by the percentage of T_REG_ (AUC = 0.82, 95% CI: 0.65 to 0.98, *P* = 0.01), DCs (AUC = 0.7983, 95% CI: 0.62 to 0.97, *P* = 0.0242), and monocytes (AUC = 0.7899, 95% CI: 0.60 to 0.98, *P* = 0.0284), and the insulin dose (AUC = 0.7971, 95% CI: 0.63 to 0.96, *P* = 0.0112). To visually examine how well these independent variables do at predicting PR, we plot the distribution of predicted probabilities for both the observed PR and non-PR groups. Looking at the violin plots for the group that experienced the PR phase, the majority of patients had predicted probabilities of being in PR above 0.5 in all cases (%T_REG_ with a median of 0.82 and mean of 0.73; %DCs with a median of 0.82 and mean of 0.77; %Monocytes with a median of 0.79 and mean of 0.77; Insulin dose with a median of 0.79 and mean of 0.72) (**Figures 9I–L**). The predicted probability of PR for the observed non-PR group was significantly lower than those of the observed PR group in all cases (*P <*0.01 for %T_REG_; *P* ≤0.05 for %DCs; *P* ≤0.05 for %Monocytes; and *P <*0.01 for Insulin dose). However, the variables did not perform as well with classifying the group of observed non-remitters, as the predicted probabilities are more uniformly distributed (%T_REG_ with a median of 0.57 and mean of 0.48; %DCs with a median of 0.58 and mean of 0.57; %Monocytes with a median of 0.63 and mean of 0.55; Insulin dose with a median of 0.49 and mean of 0.47).

### An Index-Based Model Comprising the Percentage of T_REG_, DCs, and Monocytes at T1D Onset Could Predict the PR

**Figure 10:**
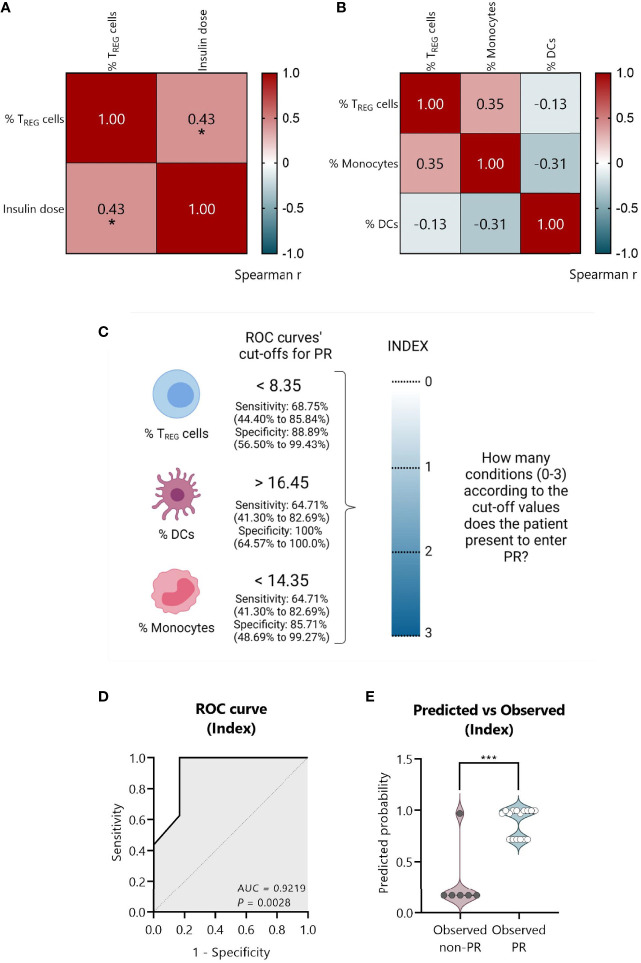
An index-based model comprising the percentage of T_REG_, DCs, and monocytes could predict PR at T1D diagnosis. **(A)** Spearman’s correlation coefficient matrix between insulin dose and the percentage of T_REG_ (*n* = 25), and **(B)** between the percentages of T_REG_, DCs, and monocytes (*n* = 22) are shown in the form of a heatmap for *r* values (blue; positive correlation; red, negative correlation). **(C)** Strategy followed to create an index comprising all the independent significant variables. From ROC curves, the best cut-off values were selected to discriminate between the remitter and the non-remitter groups for the percentage of T_REG_ (<8.35), DCs (>16.45), and monocytes (<14.35), considering both the sensitivity and the specificity with 95% CI. An index including the selected cut-off’s values was created to determine for each patient how many conditions they met for entering PR on a 0–3 scale (0, no condition; 3, all conditions), being the cut-offs the conditions that should be fulfilled. **(D)** Graphical representation of the AUC for the created index. **(E)** Violin plot showing the frequency distribution of predicted probabilities for both the observed PR (*n* = 16) and non-PR (*n* = 6) groups regarding the index. Each dot represents an individual patient. Violin plots show the median (thick dashed line) and first and third quartiles (thin dashed lines). *P ≤0.05 after Spearman’s correlation test, ***P <0.001 after 2-tailed Mann–Whitney test. *P* ≤0.05 is considered significant.

To create a full model containing simultaneously the statistically significant variables, we tested if they were monotonically related through Spearman’s tests. The percentage of T_REG_ positively correlated with the insulin dose (Spearman’s *r* = 0.43, *P* ≤0.05) (**Figure 10A**). No significant correlations were found between the percentage of T_REG_, monocytes, and DCs in the correlation matrix plot in **Figure 10B**. Thus, a model containing only independent immune-related variables was created.

First, from the ROC curves (**Figures 9E–G**) we selected the best cut-off values to discriminate between remitters and non-remitters for each of the three independent variables, considering both the sensitivity and the specificity. For the percentage of T_REG_, the best cut-off value to determine PR was <8.35 with a sensitivity of 68.75% (95% CI: 44.40 to 85.84%) and a specificity of 88.89% (95% CI: 56.50 to 99.43%); for the percentage of DCs, the best cut-off value was >16.45 with a sensitivity of 64.71% (95% CI: 41.30 to 82.69%) and a specificity of 100% (95% CI: 64.57 to 100.0%); and for the percentage of monocytes, the best cut-off value was <14.35 with a sensitivity of 64.71% (95% CI: 41.30 to 82.69%) and a specificity of 85.71% (95% CI: 48.69 to 99.27%) (**Figure 10C**).

Since the sample size is limited and to avoid problems of overfitting of the multiple variables, an index including the cut-off’s values was created to determine on a 0–3 scale how many conditions or cut-offs the patients meet for entering the PR phase, reducing in that way three variables to one. In that sense, higher values represent more conditions met (0, no condition; 3, all conditions) (**Figure 10C**). Simple logistic regression analysis was used to determine the association between the event of PR and this index, finding 12-fold increased odds for being in PR with each condition met (OR = 12.49, 95% CI: 2.350 to 283.5, *P* = 0.03) (**Table 4**). The G-test score for this model was 12.75 (*P* = 0.0004). Then, to evaluate the discriminatory ability of this model among patients who will or will not be in PR, a ROC curve was performed, presenting an AUC of 0.9219, 95% CI: 0.77 to 1.00 (*P* = 0.0028) (**Figure 10D**). Moreover, in the violin plot, the distribution of predicted probabilities for the observed PR group was far above 0.5 in all cases, being most of them around 1 (median of 0.97 and mean of 0.89). On the contrary, the distribution of predicted probabilities for the observed non-PR group was below 0.5 in all but one patient (median of 0.17 and mean of 0.30) (*P <*0.001) (**Figure 10E**).

**Table 4:** Simple logistic regression of index as a determinant of PR in pediatric patients at T1D onset and its adjustment for BMI-SDS.

Simple logistic regression							
Variable	Coefficient	SE	OR [95% CI]	|Z|	*P*-value^C^	G test	*P*-value^D^
Index^A^	2.525	1.159	12.49 [2.350 to 283.5]	2.18	0.03*	12.75	0.0004***
**Multiple logistic regression**							
Index^A^	2.669	1.335	14.43 [2.371 to 568.1]	1.999	0.05*	12.83	0.0016**
BMI-SDS^C^	−0.374	1.303	0.69 [0.043 to 10.69]	0.287	0.77		

^A^T_REG_ (%) + Monocytes (%) + DCs (%) cut-off values from independent ROC curves as conditions to be fulfilled on a 0–3 scale. ^C^P-value from OR; ^D^P-value from G test. BMI, Body Mass Index; SDS, standard deviation score; SE, standard error. *P ≤0.05, **P ≤ 0.01, ***P ≤ 0.001.

Female sex, being younger than 5 years old, and higher BMI values have been related to the risk for non-remission (13, 33). Since age and BMI are highly correlated, we decided to adjust our index for BMI-SDS, which is standardized for child age and sex. Multiple logistic regressions revealed 14-fold increased odds for being in PR with each condition met (OR = 14.43, 95% CI: 2.371 to 568.1, *P* ≤0.05), while BMI-SDS was not statistically significant (**Table 4**). Therefore, by including this clinical parameter, the positive predictive effect of the simple logistic regression is maintained at a significant level. Finally, multicollinearity was evaluated for the variables index and BMI-SDS using VIF and R^2^ with other variables. Regarding both the index and BMI-SDS, VIF was 1.002 and R^2^ was 0.0017 (**Supplemental Table 6**). Consequently, multicollinearity is not a problem in our data.

In summary, a model comprising the percentages of T_REG_, DCs, and monocytes all together in an index could predict the PR phase in children and adolescents at T1D diagnosis.

## Discussion

The PR phase is still a poorly characterized stage of T1D natural history but is of great interest given its association with better glycemic control and the consequent reduction in secondary complications (8). In this study, we have analyzed up to 52 peripheral immune cell subpopulations and different cytokines in plasma during one year from T1D onset, focusing mainly on the stage of PR. Specific alterations of this phase have been discovered in terms of the percentage of EM T lymphocytes, T_ERMA_ lymphocytes, T_REG_, neutrophils, DCs, B_REG_, and transitional T1 B lymphocytes. In addition, we have created a prediction model of PR that is based on an index that considers the percentages of T_REG_, monocytes, and DCs, and that could distinguish between remitters and non-remitters at diagnosis. Despite needing independent validation, these candidate immunological biomarkers to monitor and predict the PR corroborate that this stage is governed by both metabolic and immunological factors.

Although some studies investigating the PR phase have been reported, only a few have performed a longitudinal follow-up together with comparisons between remitter and non-remitter patients at an immunological level. To the best of our knowledge, and based on the IDAA1c index to define remission, this is the first study that prospectively monitors immune cell subsets and cytokines in children and adolescents with T1D from disease onset to the first year after diagnosis, focusing on the PR phase and comparing between remitters and non-remitters to find reliable specific biomarkers.

CD8^+^ T cells are the predominant component of insulitis in recent-onset T1D followed by macrophages and CD4^+^ T cells (34, 35). The here reported increase in the percentages of peripheral blood CD4^+^ and CD8^+^ T_ERMA_ lymphocytes and EM CD4^+^ T lymphocytes at PR could reflect their lower migration to the pancreas, resulting in reduced percentages of peripheral naïve T lymphocytes. According to these findings, increased percentages of circulating cytotoxic T lymphocytes positive for IFN-γ, T_H_1 lymphocytes, and T_H_17 lymphocytes have been found during the PR phase in comparison to the disease onset (21), and also after the first year from diagnosis for CD4^+^ and CD8^+^ T_ERMA_ lymphocytes, EM CD4^+^ T lymphocytes, and T_H_17 lymphocytes (19). Of note, islets from patients with T1D express CXCL10, a chemokine involved in autoreactive T lymphocyte recruitment, while in controls, neither CXCL10^+^ endocrine cells nor CXCR3^+^ lymphocytes were detected (36). That could partially explain the lower percentages of EM CD8^+^ T cells observed at T1D onset in the periphery (37). Also, β-cells from patients with T1D hyperexpress HLA class I, thus increasing autoantigen presentation to autoreactive CD8^+^ T cells (38), which were found within the islets showing an antigen-experienced phenotype (CD45RA^−^) (39, 40). Since the main mediators of β-cell destruction in T1D are autoreactive effector CD4^+^ and CD8^+^ T cells, and supposing that EM T lymphocytes are acting less *in situ*, their increase in the periphery during PR could reflect an attempt at immunoregulation and β-cell recovery. Interestingly, a recent longitudinal study (24) described an association of the PR phase with the restoration of the programmed cell death-1/programmed death-ligand 1 axis on T cells, suggesting a mechanism of immunoregulation that did not occur in non-remitter patients.

On the other hand, homeostasis between T_REG_ and effector T lymphocytes is crucial for the induction and maintenance of peripheral tolerance. The prediabetic phase in the natural history of T1D is indeed very heterogeneous; it can last from months to years, and different factors influence the fact of presenting overt T1D, like the number and titers of autoantibodies. Evidence supports that having higher frequencies of insulin-specific T_REG_ is associated with a slow progression to clinically symptomatic T1D (41), but that at this time-point, T_REG_ are dysfunctional, thus contributing directly to disease development [reviewed in (42)]. However, the dominance of T_REG_ over effector T cells may contribute to PR occurrence (32). In fact, islet-specific CD8^+^ T cells with an exhaustion-like profile identify patients with slow T1D progression after onset (43). Until recently, it was generally accepted that the overall frequency of peripheral blood CD4^+^FoxP3^+^ T_REG_ is unaltered in patients with T1D (44–46). Nonetheless, contradictory data on T_REG_ have been reported, probably because of the different ways that these cells can be identified. Here, we found that CD4^+^CD25^+^CD127^−/low^ and memory T_REG_ are decreased in percentage at PR in comparison to patients without PR. One possible explanation is that these cells could recover their impaired function with the rapid rectification of hyperglycemia after T1D onset, being more active in secondary lymphoid organs and the target tissue. Using the same markers, Fitas et al. (21) found only a decrease in T_REG_ after a year from T1D onset, but not during the PR phase, while we previously found an increase in activated T_REG_ one year after diagnosis (19). Other studies investigating the association of T lymphocytes and PR found that its length positively correlates with the high frequency of activated T_REG_ (18) and CD4^+^CD25^+^CD127^hi^ T cells (17) at disease onset. Furthermore, IL-10-dependent regulatory CD4^+^ T lymphocyte pathways are involved in long-term remission of T1D (15), and islet-specific IL-10^+^ immune responses but not CD4^+^CD25^+^FoxP3^+^ cells at diagnosis predict glycemic control. In fact, peripheral antigen-specific T_REG_ were diminished during PR in comparison to diagnosis (22). We found this trend for total T_REG_ cells in most of our remitter patients except four. Those patients presented a similar behavior to non-remitters, who presented increased percentages of T_REG_ cells from disease onset. Interestingly, they are the youngest within the PR group. Since pediatric patients diagnosed before age 7 may have a more aggressive form of T1D, that could be related to the observed changes in T_REG_ cells (47). In conclusion, further characterization of T_REG_ subsets in terms of phenotype and function needs to be addressed to dissect their role during PR.

Innate immune cells are crucial players in the development and progression of T1D and multiple interactions occur between them and lymphocytes (48). Here, neutrophils and DCs were found to be altered during PR. Neutrophils are present in the insulitis before diagnosis, and they continue infiltrating the pancreas as the disease progresses, having a direct pathogenic role (49). Previous results showed a reduction in neutrophil counts at diagnosis that is associated with a poor endocrine pancreatic function (50, 51). Here, we did find a reduction in CD16^+^ neutrophil absolute counts in non-remitter patients both at 8 and 12 months after the diagnosis when compared to remitter patients. Peripheral neutrophils mainly express a rather atypical CD16, the FcγRIIIB, which can trigger neutrophil activation. The possible explanations for the decreased CD16^+^ neutrophil are 1) the greater activation or apoptosis that would involve FcγRIIIB proteolytic cleavage, 2) tissue detainment, or 3) abnormal maturation (51–53). As hyperglycemia is linked to neutrophil dysfunction, non-remitters are more susceptible to present those impairments. On the other hand, DCs play a pivotal role in modulating T cell responses by altering the balance between tolerance and autoimmunity (54). We found that patients at PR present lower percentages of DCs than non-remitter patients. Previous research in pediatric patients found impaired functionality, percentages, and/or numbers in total DCs, mDCs, and pDCs both at T1D onset and one year after diagnosis (55–57). One explanation could be the previously described reduced production of DCs from monocytes in T1D (58), although our data on the DC numbers do not fit well with those results. Also, inflammation causes CCL2 release from islets, prompting translocation of CCR2^+^ DCs from circulation to inflamed tissues, thus causing a reduction in DCs counts and their CCR2 expression in peripheral blood (56). Since the amelioration of hyperglycemia could contribute to the recovery of the immunosuppressive properties of DCs during the PR phase (59), tolerogenic DCs expressing CCR2 could be more active in the target tissue, diminishing in peripheral blood and prompting T_REG_ responses.

In addition, after 12 months of follow-up, only patients that experienced PR showed increased levels of B_REG_ and transitional T1 B lymphocytes. According to our results, other studies in new-onset T1D have not found alterations in other B cell subsets (37, 60), while transitional B cells and B_REG_ increased in pediatric patients one year after diagnosis (19). Both B_REG_ and transitional B lymphocytes have immunoregulatory properties and can inhibit effector T cell proliferation (61, 62). Some results suggest that B cell subsets could play a role in T1D pathogenesis, for instance, both the percentage of IL-10^+^ B_REG_ and IL-10^+^ immature transitional B cells were significantly lower in patients with T1D at diagnosis than in controls and patients with worse glycemic control (63, 64). Regarding transitional B cells, these are classified in T1 and T2 subsets, having T1 cells greater immunosuppressive properties. Therefore, the here reported higher ratio of T1/T2 cells and the increased counts of B_REG_ after a year of follow-up for remitter patients could reflect an immunoregulatory attempt.

Cytokines orchestrate multiple interactions between β cells and immune cells. Here, none of the analyzed cytokines showed significant differences between remitters and non-remitters, which would limit their use as biomarkers of remission or T1D progression. However, we found increased levels of IL-17A, a relevant cytokine in T1D (65), at disease onset, and a trend to reduce this cytokine in remitter patients. IL-17A expression is upregulated in the pancreas of both humans and animal models (44, 66), and among other effects, it recruits and activates neutrophils (67). Because IL-23 and/or TGF-β plus IL-6 drive the production of IL-17 by T cells, the here reported IL-17A increase together with the higher levels of TGF-β and IL-6 could reflect the inflammatory state at diagnosis, which is less evident at PR. In fact, we found a positive correlation between IL-6 and IL-17A at T1D onset, whereas this correlation was negative in controls (data not shown). In a previous non-longitudinal study, low TGF-β levels were a feature of PR (19), which has not been confirmed in this longitudinal study. While none of the analyzed cytokines were predictive of PR, in a prospective study of T1D, lower levels of IL-10, IFN-γ, and IL-1R1 at diagnosis were associated with remission (26). In summary, the use of cytokines as T1D progression markers does not seem to have enough power to correctly distinguish PR.

Because the efficacy of immunotherapies depends on the time of intervention, mathematical models for predicting disease progression (68) and PR are important tools to improve therapeutic strategies. Regarding metabolism, previous studies have shown associations between metabolic and clinical parameters and the PR phase. In our bivariate analysis, only the lower insulin dose at T1D onset could predict remission. Although not significant, and according to previous data, we found that patients with higher BMI and older age at diagnosis are more prone to experience remission (69). In general, children experience remission to a higher extend than adults, and within the first group, the probability of remaining in remission is greater as the onset age increased (70). Different regression analyses showed that higher pH and bicarbonate levels, higher BMI, male sex, and lower HbA1c, insulin dose, and numbers of islet antibodies at T1D onset are predictors of remission (13, 14, 70–72). However, the prediction of remission using immunological data is rather scarce. Unlike IFN-γ concentration, elevated levels of IL-10 and IL-6 in serum are positively associated with remission (22, 23), and the absence of IL-4, TNF-α, IL-10, and IL-13 detection is positively correlated with its length (21). Also, CD4^+^CD45RO^+^ T cells, activated T_REG_, CD4^+^CD25^+^CD127^hi^ T cells, and apoptotic T_REG_ are reportedly associated with remission (17, 18, 73). Here, we found that the percentages of T_REG_, monocytes, and DCs at T1D onset are independent predictors of the PR phase. T_REG_ were also positively correlated with the insulin dose, highlighting the crucial role of immunometabolism interactions during T1D and the mechanisms rectifying glucose toxicity in the process of β-cell protection. The bivariate analysis of the generated index, which includes the cell percentages, showed an even higher ability to distinguish remitters from non-remitters. When the index was adjusted for possible confounders, only it remained a significant predictor of PR. In summary, we propose a novel immune-based predictive model of remission, although it must be further validated in a larger cohort of patients.

We are aware of the limitations of the presented study. First, the number of individuals studied lowers the statistical power; however, such data are very hard to obtain. Another weak point of our cohort is the wide range of ages (from 4 to 18) and the variability that this entails regarding the physiological changes that the subjects undergo (i.e., puberty). Whereas variations in the levels of immune cell subpopulations over one year in healthy pediatric or adult subjects are subtle or inexistent (74, 75), changes are observed within longer tracking periods (76). In that sense, we performed correlations between age and the percentage or absolute counts of the different analyzed immune cell subsets both in controls and patients at T1D onset. We observed that the levels of transitional B cells and CD8^+^ T_EMRA_ cells are negatively correlated with age and that the levels of EM and CM CD4^+^ T cells are positively correlated (data not shown). Since our longitudinal data showed an increase in transitional B cells after one year of follow-up and a decrease in the percentage of EM CD4^+^ T cells, these changes are likely to be a consequence of T1D progression. Although the usage of peripheral blood as a source of biomarkers is a non-invasive technique, immune cells are tightly regulated by their generation in primary lymphoid organs and their migration to tissues, their changes in the periphery being difficult to interpret. The analysis of the target organ and further studies on the functionality of immune cells (i.e., cytokine production)—including antigen-specific T cells—are needed to correlate changes in the periphery with the events occurring in the pancreas. Moreover, a larger sample size in future studies is needed to validate the here proposed biomarkers for the PR phase. Since limited amount of blood was obtained from pediatric patients, autoantibodies to insulin (IAA) have not been explored. However, due to its β-cell specificity, the baseline titer of IAA could be of interest for the study of the PR phase. Still focusing on this stage, we are not evaluating its length, which could be related to the persistence and activity of certain immune cells. The strengths of the study include the longitudinal picture of T1D early stages, the characterization of the PR phase versus non-remission by a wide range of immune subpopulations and cytokines, and the use of IDAA1c of ≤9 to define PR, an index that includes glycemic control and insulin doses, that correlates with C-peptide levels, and that has been validated in a large cohort of young patients with T1D (77).

In conclusion, the PR phase is not only accompanied by changes in different metabolic parameters, but also by changes in immune cells and molecules. These alterations could be potential monitoring and predictive biomarkers useful for patient stratification in clinical trials and the identification of patients with better glycemic control that will enable a more personalized therapeutic management.

## Data Availability Statement

The original contributions presented in the study are included in the article/**Supplementary Material**. Further inquiries can be directed to the corresponding author.

## Ethics Statement

The studies involving human participants were reviewed and approved by the Committee on the Ethics of Research of the Germans Trias i Pujol University Hospital and Parc Taulí University Hospital. Written informed consent to participate in this study was provided by the participants’ legal guardian/next of kin.

## Author Contributions

LGM, DPB, SRF, and MVP designed the experiments. FV, MM, AV, JP, RC, LC, and JB selected the patients, obtained the samples, and/or determined autoantibodies. LGM and DPB performed the experiments. LGM, DPB, and JMCA analyzed the data. LGM and MVP wrote the manuscript. All authors listed have made a substantial, direct, and intellectual contribution to the work and approved it for publication.

## Funding

This study has been funded by Instituto de Salud Carlos III through the project PI18/00436 (Co-funded by European Regional Development Fund ‘A way to make Europe’), and by DiabetesCero Foundation. LGM is supported by the Generalitat de Catalunya (PERIS PIF-Salut Grant No. SLT017/20/000049). This work has been supported by positive discussion through Consolidated Research Group #2017 SGR 103, AGAUR, Generalitat de Catalunya.

## Conflict of Interest

Unrelated to the work herein presented, MVP holds a patent that relates to immunotherapy for T1D and is co-founder of Ahead Therapeutics SL, and SRF is part-time employed at Ahead Therapeutics S.L. The other authors declare that the research was conducted in the absence of any commercial or financial relationships that could be construed as a potential conflict of interest.

## Publisher’s Note

All claims expressed in this article are solely those of the authors and do not necessarily represent those of their affiliated organizations, or those of the publisher, the editors and the reviewers. Any product that may be evaluated in this article, or claim that may be made by its manufacturer, is not guaranteed or endorsed by the publisher.

## Abbreviations

AUC, Area Under the Curve; BMI, Body Mass Index; B_REG_, Regulatory B Cells; CM, Central Memory; DCs, Dendritic Cells; EM, Effector Memory; GAD65, Glutamic Acid Decarboxylase 65; HbA1c, glycated hemoglobin; IA-2, Insulinoma Antigen-2; IDAA1c, Insulin-Dose Adjusted HbA1c; mDCs, Myeloid Dendritic Cells; pDCs, Plasmacytoid Dendritic Cells; PR, Partial Remission; ROC, Receiver Operating Characteristic; SDS, standard deviation score; T1D, Type 1 Diabetes; TEMRA, terminally differentiated effector memory T; T_REG_, Regulatory T Cells; VIF, Variance Inflation Factors; ZnT8, Zinc Transporter 8.
