# Corrigendum: Candidate biomarkers for the prediction and monitoring of partial remission in pediatric type 1 diabetes

**DOI:** 10.3389/fimmu.2024.1439643

**Published:** 2024-06-11

**Authors:** Laia Gomez-Muñoz, David Perna-Barrull, Josep M. Caroz-Armayones, Marta Murillo, Silvia Rodriguez-Fernandez, Aina Valls, Federico Vazquez, Jacobo Perez, Raquel Corripio, Luis Castaño, Joan Bel, Marta Vives-Pi

**Affiliations:** ^1^ Immunology Department, Germans Trias i Pujol Research Institute and University Hospital, Autonomous University of Barcelona, Badalona, Spain; ^2^ Department of Political and Social Sciences, Health Inequalities Research Group (GREDS-EMCONET), Pompeu Fabra University, Barcelona, Spain; ^3^ Johns Hopkins University–Pompeu Fabra University Public Policy Center, Barcelona, Spain; ^4^ Pediatrics Department, Germans Trias i Pujol Research Institute and University Hospital, Autonomous University of Barcelona, Badalona, Spain; ^5^ Endocrinology Department, Germans Trias i Pujol Research Institute and University Hospital, Autonomous University of Barcelona, Badalona, Spain; ^6^ Pediatric Endocrinology Department, Parc Taulí Hospital Universitari, Institut d’Investigació i Innovació Parc Taulí I3PT, Autonomous University of Barcelona, Sabadell, Spain; ^7^ Cruces University Hospital, Biocruces Bizkaia Research Institute, UPV/EHU, CIBERDEM, CIBERER, Endo-ERN, Bilbao, Spain

**Keywords:** type 1 diabetes (T1D), partial remission phase, honeymoon, biomarkers, pediatrics, prediction model, immune cell subpopulations, autoimmunity

In the published article, there was an error in the **Funding** statement. One of the funding bodies was incorrectly listed as “the Spanish Government (FIS PI18/00436)”. The correct **Funding** statement appears below.

