# Supplementary material for: Candidate Biomarkers for the Prediction and Monitoring of Partial Remission in Pediatric Type 1 Diabetes
Source: Front Immunol. 2022 Feb 23;13:825426. doi: 10.3389/fimmu.2022.825426 (PMC8904370; doi:10.3389/fimmu.2022.825426)
Supplement: Supplementary file 1 [file DataSheet_1.docx]

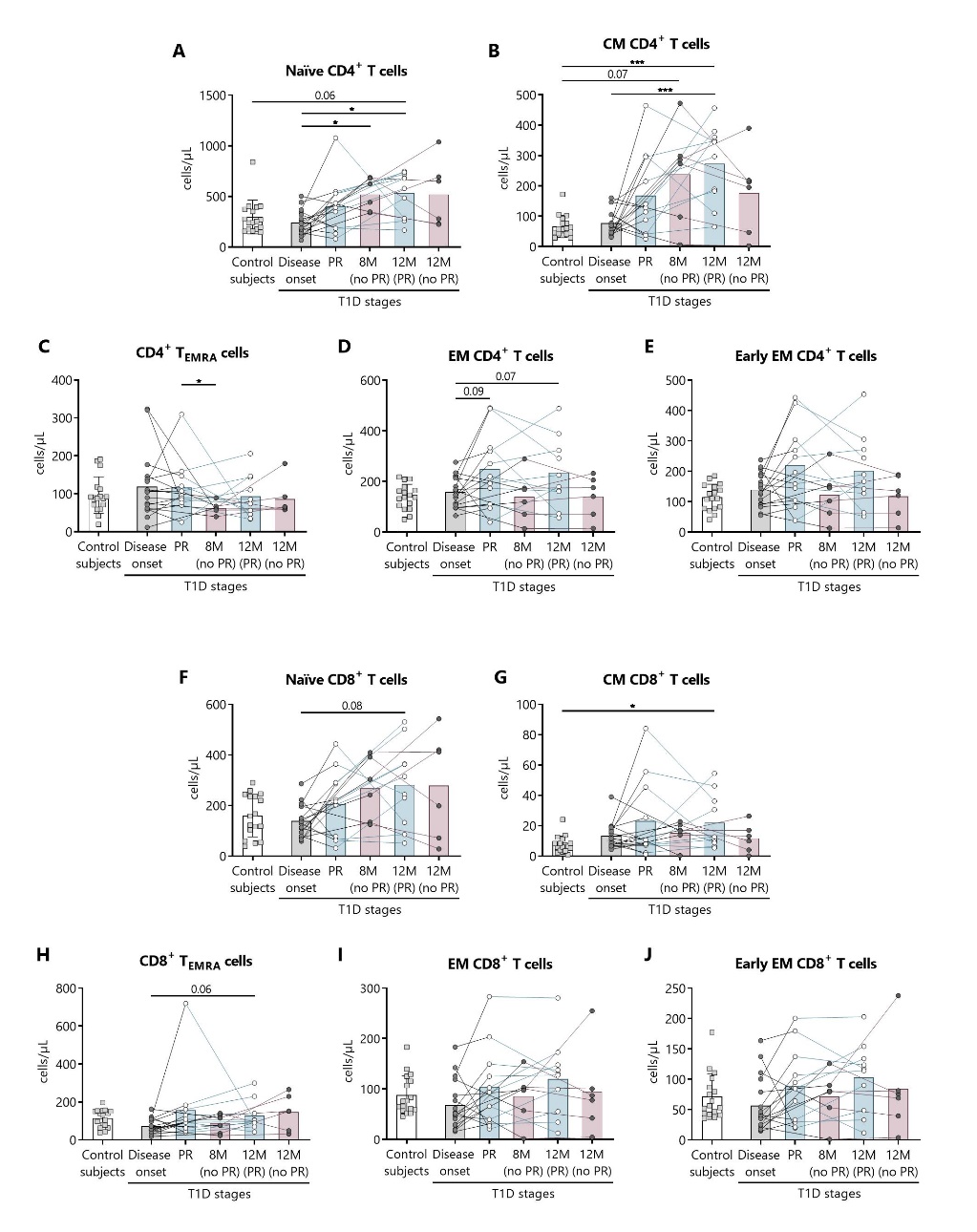
Supplemental Figure 1. Absolute counts of CD4^+^ and CD8^+^ T lymphocyte subsets are altered at the initial stages of T1D. For CD4^+^ and CD8^+^ T lymphocytes, respectively, the absolute counts (cells/µL) of (A and F) naïve T lymphocytes, (B and G) central memory (CM) T lymphocytes, (C and H) terminally differentiated effector memory (EMRA) T lymphocytes, (D and I) effector memory (EM) T lymphocytes, and (E and J) early EM T lymphocytes were determined in peripheral blood of control subjects and patients with T1D at different time-points. Squares represent control subjects (n=17) (white bar), and patients are represented by light grey dots at disease onset (n=17) (grey bar), white dots during the partial remission (PR) phase (n=11) and for remitter patients at 12 months (12M PR) (n=10) (blue bars), and dark grey dots for non-remitter patients at 8 months (8M no PR) (n=6) and 12 months (12M no PR) (n=6) (pink bars). Bar graphs show mean absolute count values. Each symbol represents an individual patient. Lines link the same patient throughout the different time-points. *P <0.05, ***P <0.001 after mixed effects model with Tukey’s post-hoc test for longitudinal data, Kruskal-Wallis with Dunn's post-hoc test for comparisons between control subjects and the different T1D time-points, or 2-tailed Mann-Whitney test for comparisons between two unpaired groups of data. P ≤0.05 is considered significant.


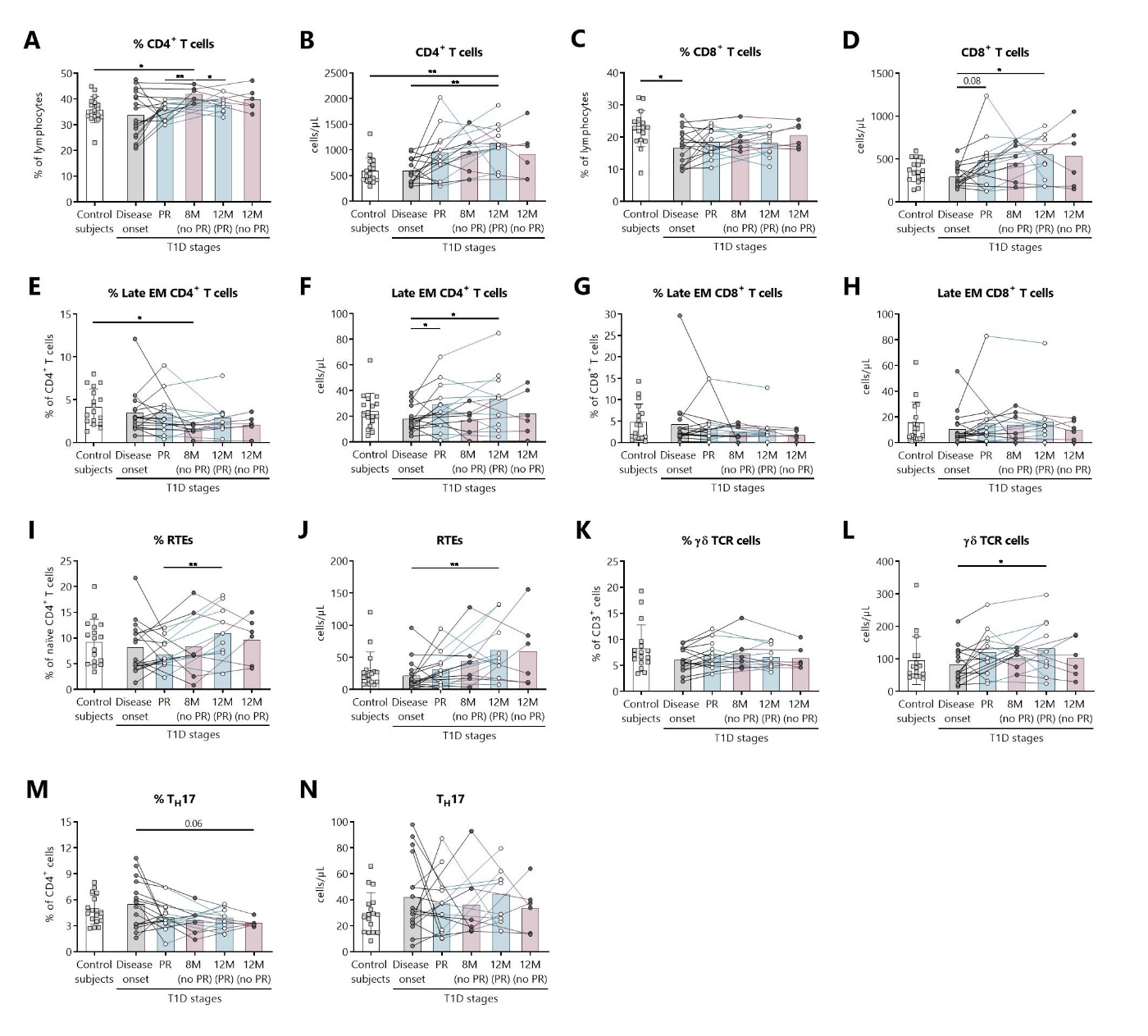
Supplemental Figure 2. Absolute counts and percentages of T lymphocyte subsets do not differ between remitters and non-remitters, except for the total percentage of CD4^+^ T lymphocytes. Percentages (%) and absolute counts (cells/µL) of (A and B) CD4^+^ T lymphocytes, (C and D) CD8^+^ T lymphocytes, (E and F) late effector memory (EM) CD4^+^ T lymphocytes, (G and H) late EM CD8^+^ T lymphocytes, (I and J) recent thymic emigrants (RTEs), (K and L) γδ TCR T lymphocytes, and (M and N) T_H_17 lymphocytes were determined in peripheral blood of control subjects and patients with T1D at different time-points. Squares represent control subjects (n=17) (white bar), and patients are represented by light grey dots at disease onset (n=16-17) (grey bar), white dots during the partial remission (PR) phase (n=11) and for remitter patients at 12 months (12M PR) (n=9-10) (blue bars), and dark grey dots for non-remitter patients at 8 months (8M no PR) (n=6) and 12 months (12M no PR) (n=6) (pink bars). Bar graphs show mean percentage or absolute count values. Each symbol represents an individual patient. Lines link the same patient throughout the different time-points. *P <0.05, **P <0.01 after mixed effects model with Tukey’s post-hoc test for longitudinal data, Kruskal-Wallis with Dunn's post-hoc test for comparisons between control subjects and the different T1D time-points, or 2-tailed Mann-Whitney test for comparisons between two unpaired groups of data. P ≤0.05 is considered significant.


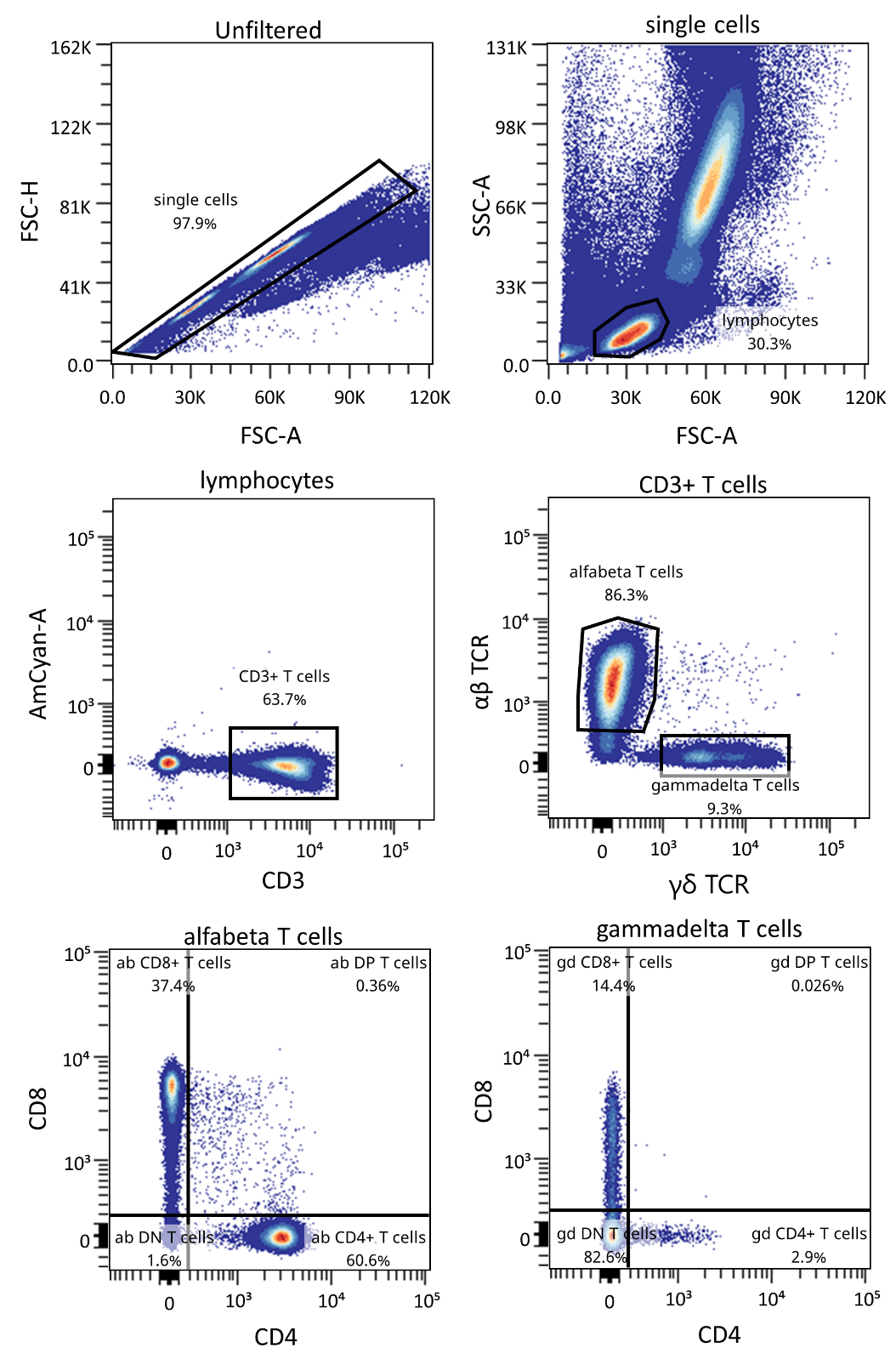


Supplemental Figure 3. Representative gating strategy for the TCR panel. Gating strategy used to analyze the percentages of the αβ and γδ TCR of CD4^+^, CD8^+^, CD4^+^CD8^+^ (DP), and CD4^-^CD8^-^ (DN) T cells.


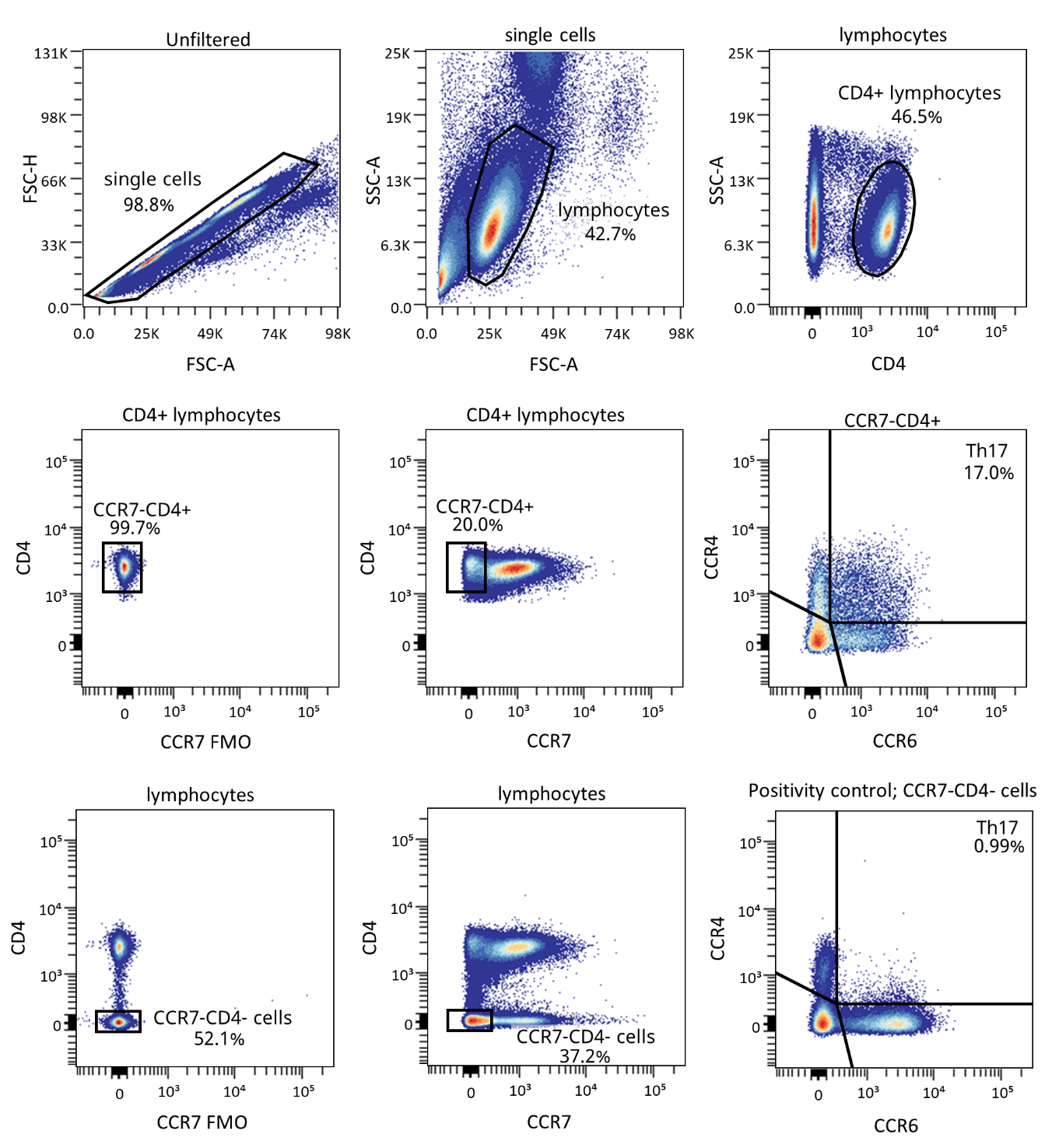


Supplemental Figure 4. Representative gating strategy for the T_H_17 cells panel. Gating strategy used to analyze the percentages of the T_H_17 cells based on the expression of the markers CD4, CCR7, CCR4 and CCR6. FMO control for CCR7 and positivity control for CCR4 vs CCR6 using CCR7^-^CD4^-^ lymphocytes as an internal reference population.


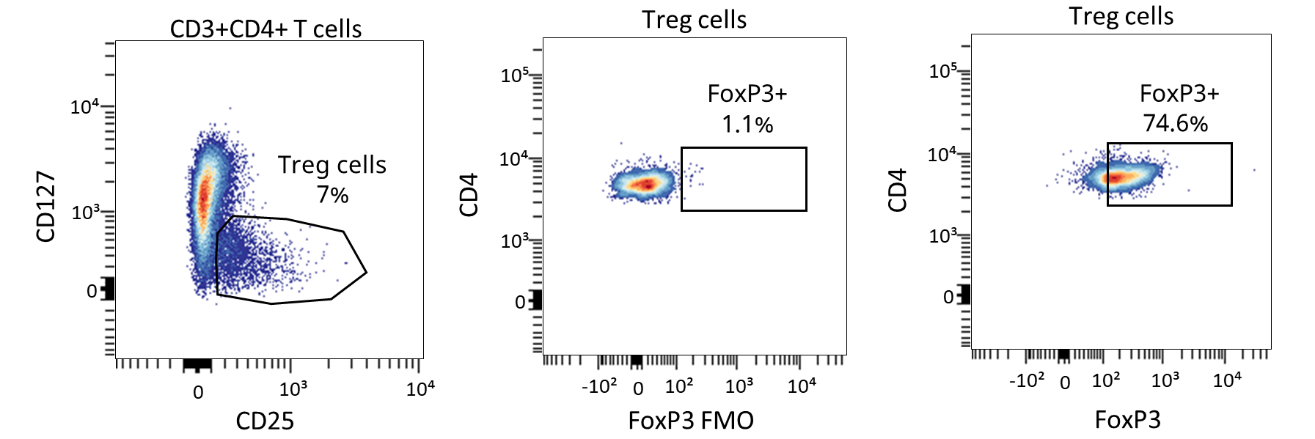


Supplemental Figure 5. Representative gating strategy for FoxP3. Gating strategy used to analyze the percentages of CD25^+^CD127^-/low^ Tregs that express FoxP3.


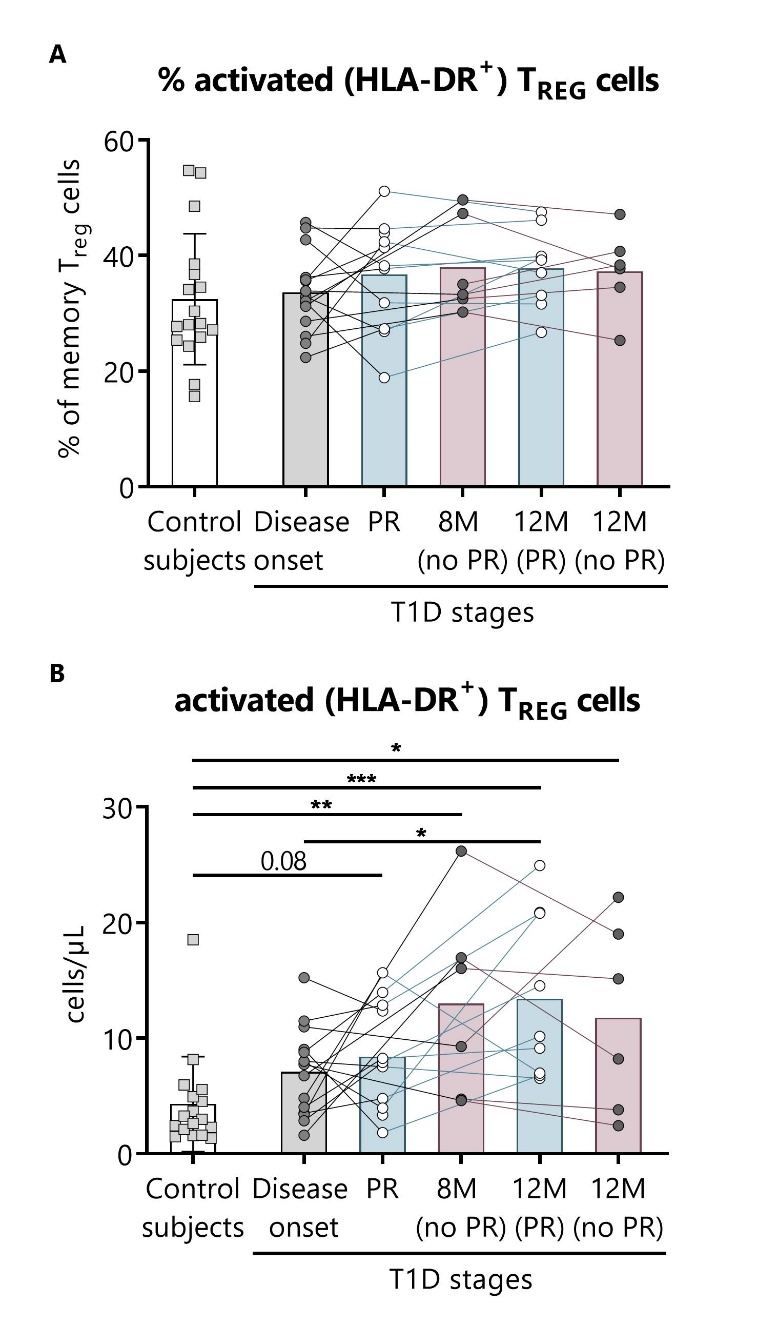


Supplemental Figure 6. Absolute counts of activated regulatory T lymphocytes, but not the percentage, are altered at the initial stages of T1D. (A) Percentages (%) and (B) concentrations (cells/µL) of activated regulatory T cells (T_REG_) were determined in peripheral blood of control subjects and patients with T1D at different time-points. Squares represent control subjects (n=17) (white bar), and patients are represented by light grey dots at disease onset (n=15) (grey bar), white dots during the partial remission (PR) phase (n=11) and for remitter patients at 12 months (12M PR) (n=9) (blue bars), and dark grey dots for non-remitter patients at 8 months (8M no PR) (n=6) and 12 months (12M no PR) (n=6) (pink bars). Bar graphs show mean percentage or absolute count values. Each symbol represents an individual patient. Lines link the same patient throughout the different time-points. *P <0.05, **P <0.01, ***P <0.001 after mixed effects model with Tukey’s post-hoc test for longitudinal data, or Kruskal-Wallis with Dunn's post-hoc test for comparisons between control subjects and the different T1D time-points. P ≤0.05 is considered significant.


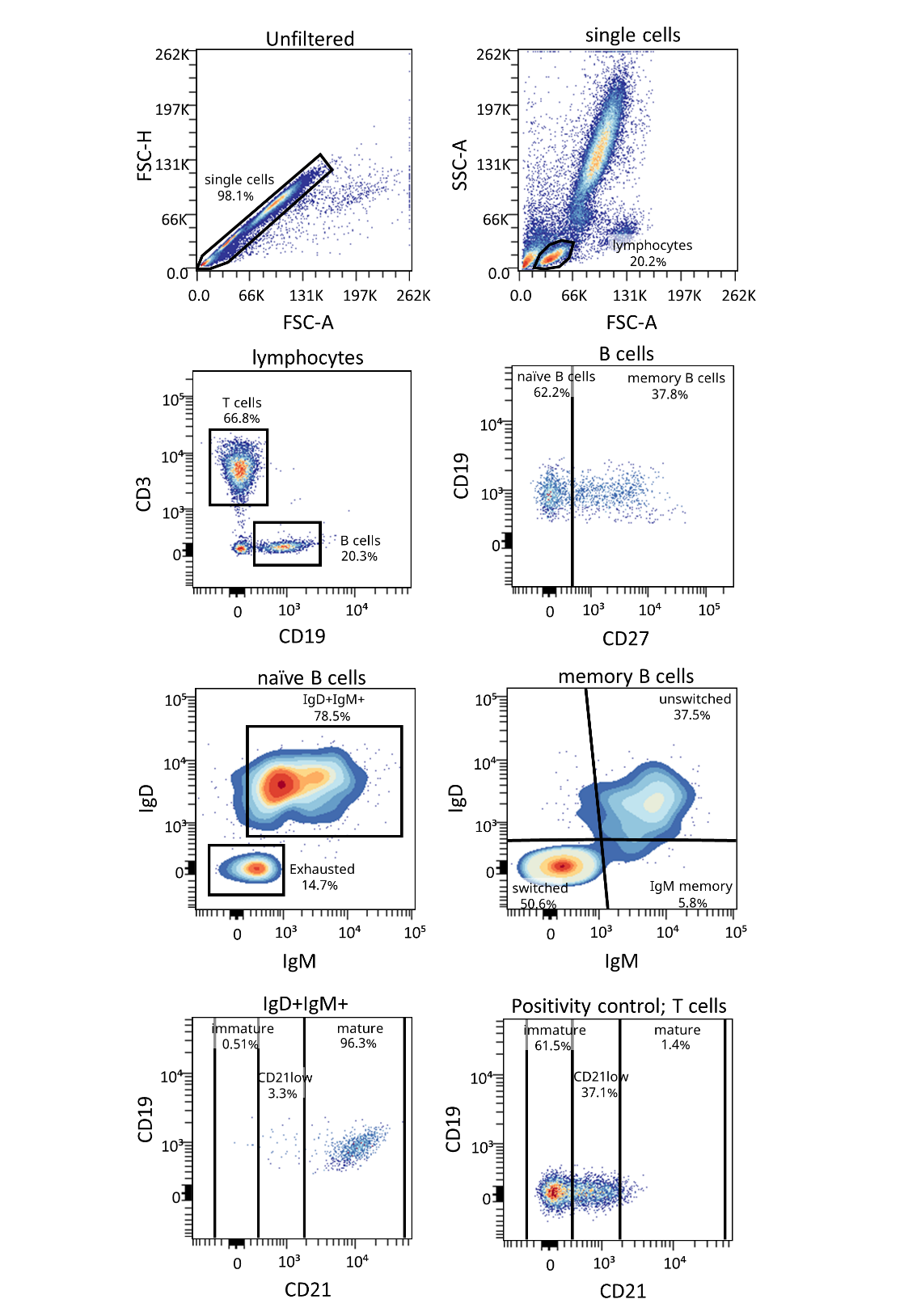


Supplemental Figure 7. Representative gating strategy for the B lymphocyte maturation stages panel. Gating strategy used to analyze the percentages and absolute counts of the different naïve and memory B cell subsets based on the expression of the markers CD19, CD27, IgM, IgD, and CD21. Positivity control for CD19 vs CD21 using CD3^+^ T lymphocytes as an internal reference population.


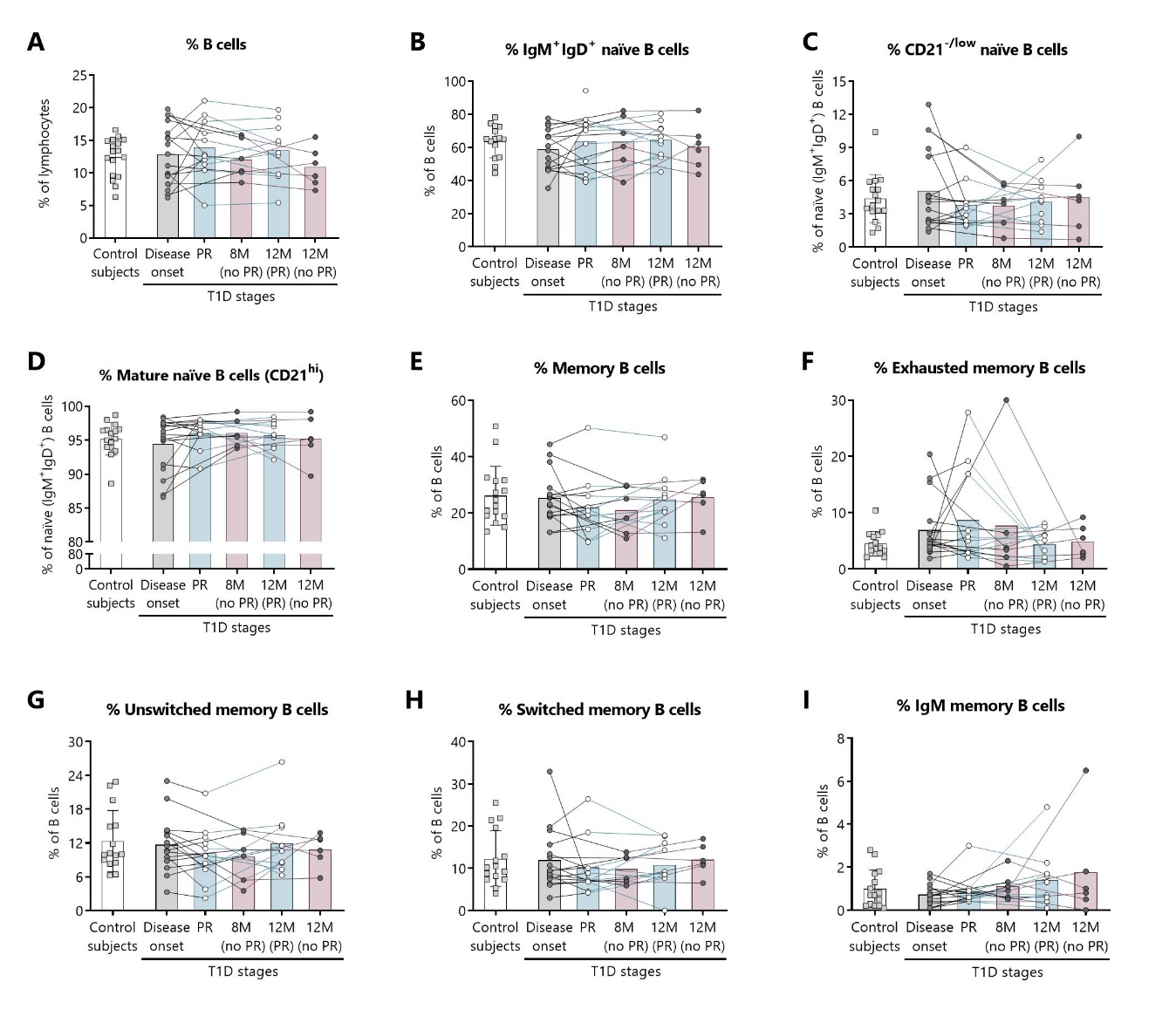
Supplemental Figure 8. The percentage of different maturation stages of B lymphocytes is not altered at the initial stages of T1D. Percentages (%) of (A) B lymphocytes, (B) IgM^+^IgD^+^ naïve B lymphocytes, (C) CD21^-/low^ naïve B lymphocytes, (D) mature naïve B lymphocytes, (E) memory B lymphocytes, (F) exhausted memory B lymphocytes, (G) unswitched memory B lymphocytes, (H) switched memory B lymphocytes, and (I) IgM memory B lymphocytes were determined in peripheral blood of control subjects and patients with T1D at different time-points. Squares represent control subjects (n=15-17) (white bar), and patients are represented by light grey dots at disease onset (n=16-17) (grey bar), white dots during the partial remission (PR) phase (n=11) and for remitter patients at 12 months (12M PR) (n=10) (blue bars), and dark grey dots for non-remitter patients at 8 months (8M no PR) (n=6) and 12 months (12M no PR) (n=6) (pink bars). Bar graphs show mean percentage values. Each symbol represents an individual patient. Lines link the same patient throughout the different time-points. Data analyzed by mixed effects model with Tukey’s post-hoc test for longitudinal data, Kruskal-Wallis with Dunn's post-hoc test for comparisons between control subjects and the different T1D time-points, or 2-tailed Mann-Whitney test for comparisons between two unpaired groups of data. P ≤0.05 is considered significant.


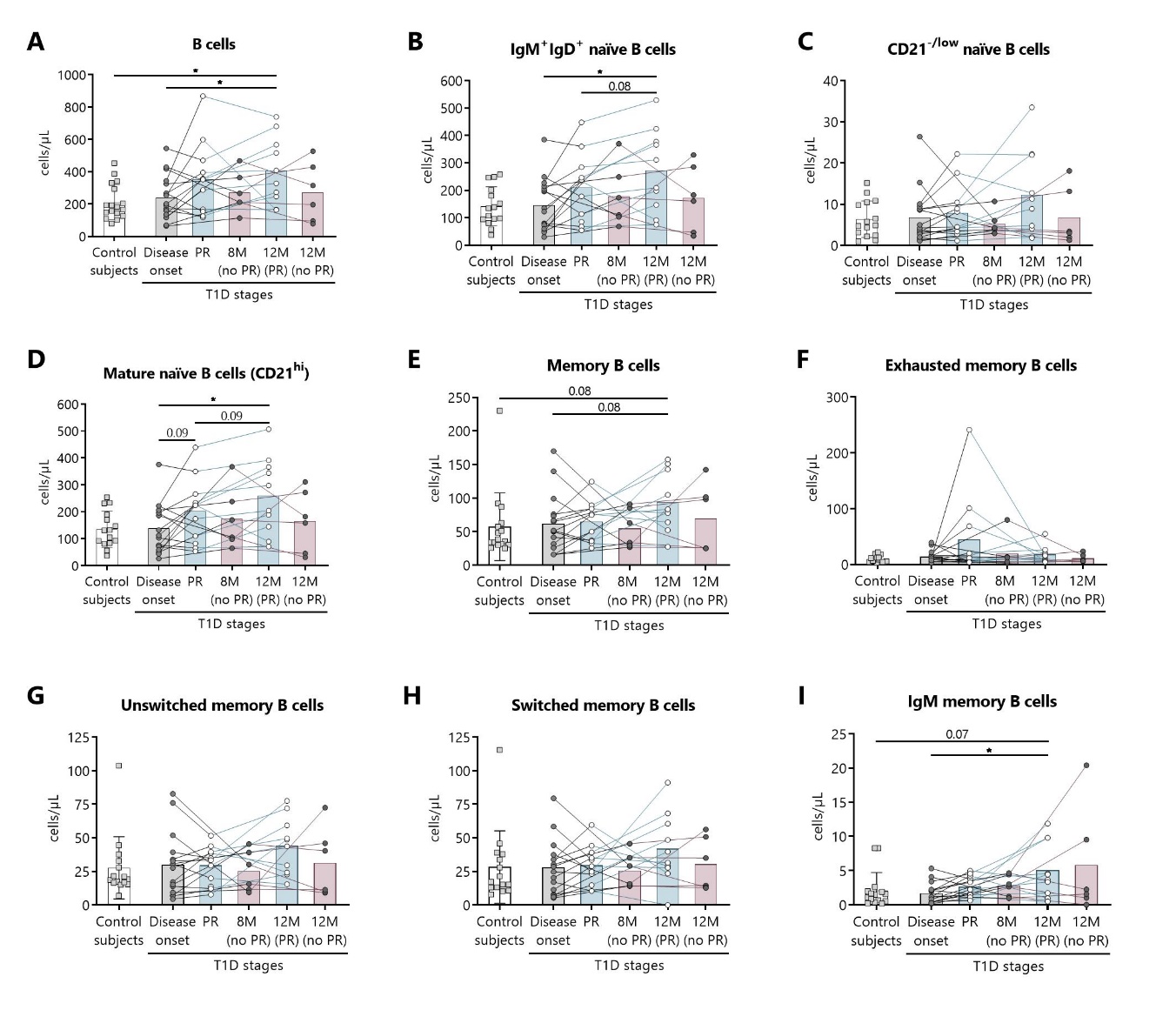
Supplemental Figure 9. Absolute counts of B lymphocytes at different maturation stages barely differ between the initial stages of T1D. Absolute counts (cells/µL) of (A) B lymphocytes, (B) IgM^+^IgD^+^ naïve B lymphocytes, (C) CD21^-/low^ naïve B lymphocytes, (D) mature naïve B lymphocytes, (E) memory B lymphocytes, (F) exhausted memory B lymphocytes, (G) unswitched memory B lymphocytes, (H) switched memory B lymphocytes, and (I) IgM memory B lymphocytes were determined in peripheral blood of control subjects and patients with T1D at different time-points. Squares represent control subjects (n=15-17) (white bar), and patients are represented by light grey dots at disease onset (n=16-17) (grey bar), white dots during the partial remission (PR) phase (n=11) and for remitter patients at 12 months (12M PR) (n=10) (blue bars), and dark grey dots for non-remitter patients at 8 months (8M no PR) (n=6) and 12 months (12M no PR) (n=6) (pink bars). Bar graphs show mean absolute count values. Each symbol represents an individual patient. Lines link the same patient throughout the different time-points. *P <0.05 after mixed effects model with Tukey’s post-hoc test for longitudinal data, or Kruskal-Wallis with Dunn's post-hoc test for comparisons between control subjects and the different T1D time-points. P ≤0.05 is considered significant.


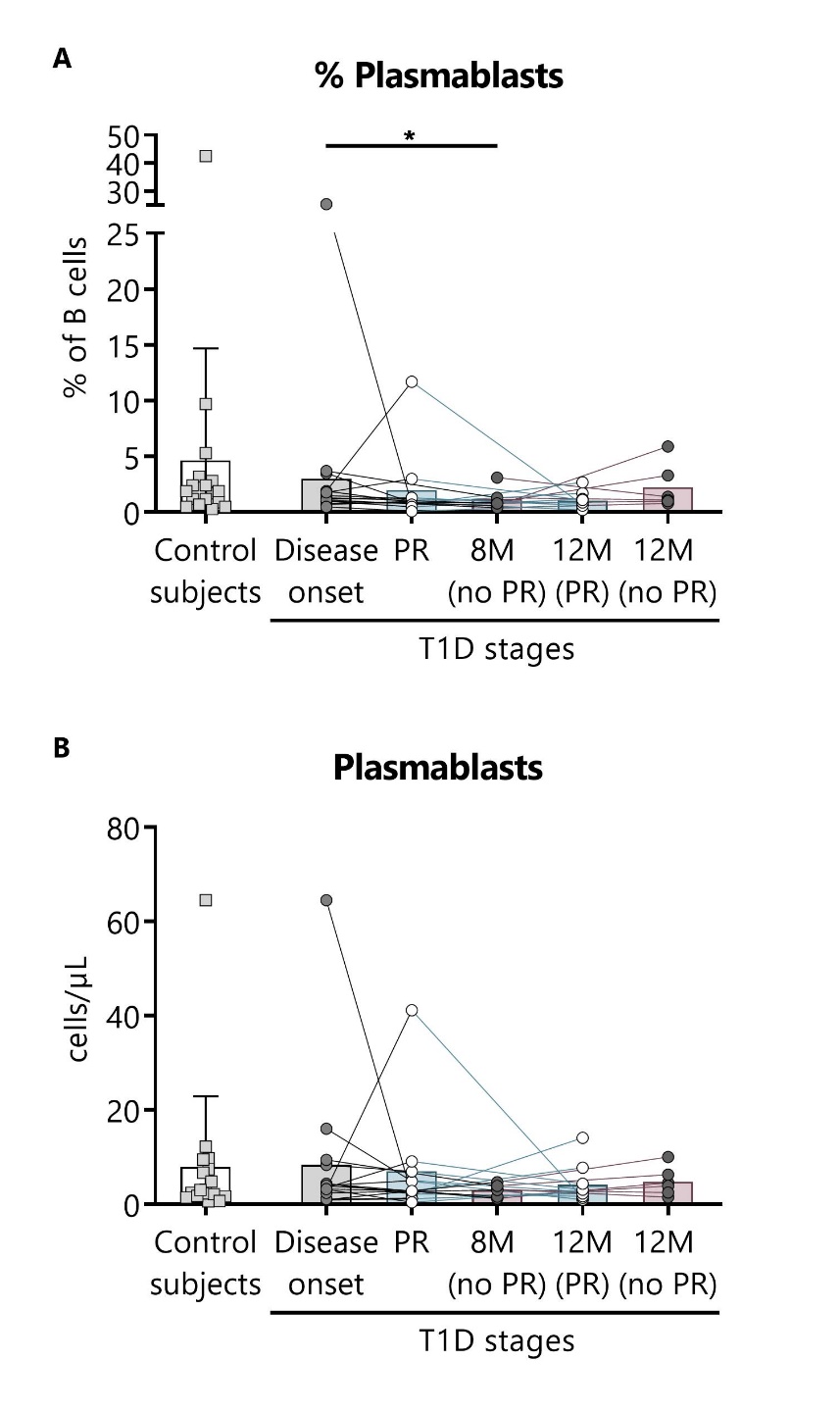


Supplemental Figure 10. The amount of plasmablasts do not differ between remitters and non-remitters patients with T1D. (A) Percentage (%) and (B) concentration (cells/µL) of plasmablasts were determined in peripheral blood of control subjects and patients with type 1 diabetes (T1D) at different time-points. Squares represent control subjects (n=17) (white bar), and patients are represented by light grey dots at disease onset (n=16) (grey bar), white dots during the partial remission (PR) phase (n=11) and for remitter patients at 12 months (12M PR) (n=10) (blue bars), and dark grey dots for non-remitter patients at 8 months (8M no PR) (n=6) and 12 months (12M no PR) (n=6) (pink bars). Bar graphs show mean percentage or absolute count values. Each symbol represents an individual patient. Lines link the same patient throughout the different time-points. *P <0.05 after mixed effects model with Tukey’s post-hoc test for longitudinal data. P ≤0.05 is considered significant.


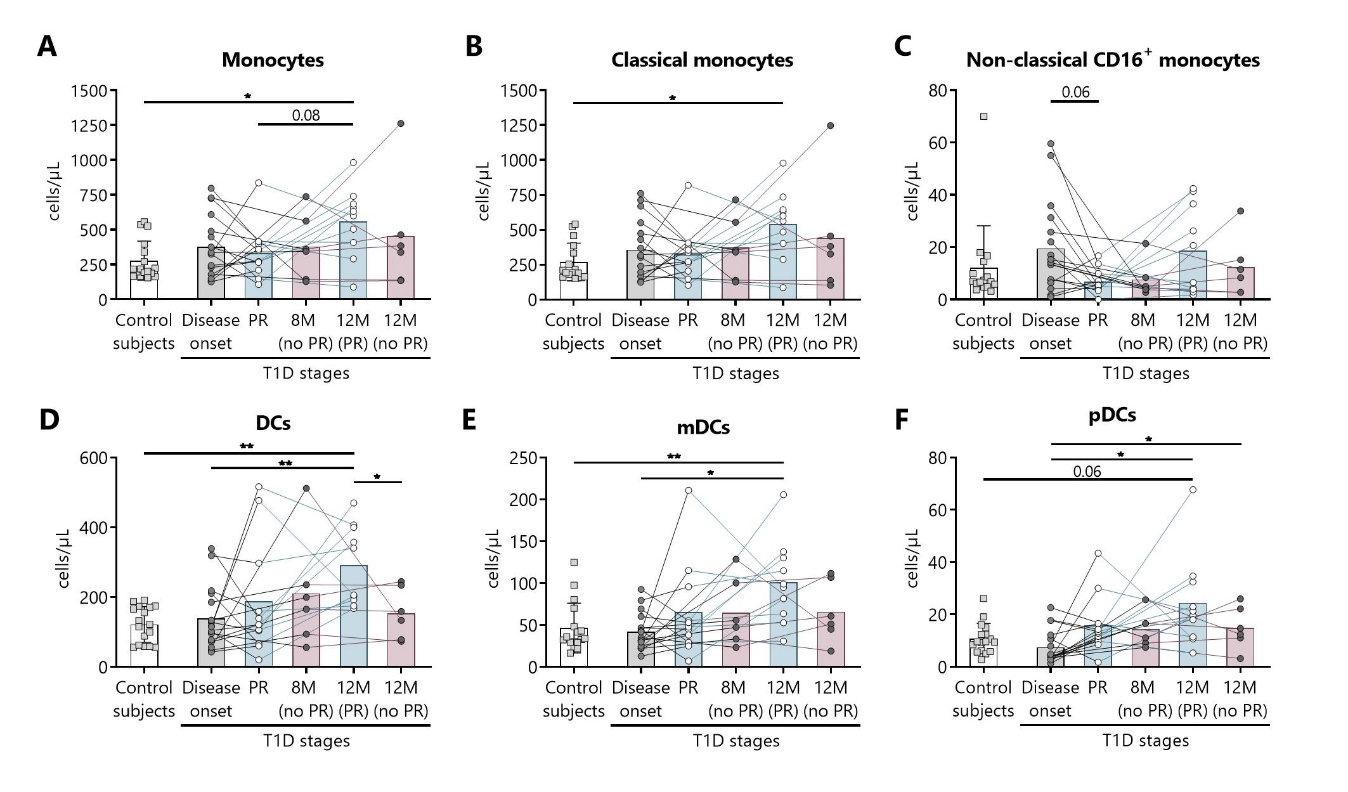
Supplemental Figure 11. Absolute counts of monocytes and dendritic cells are altered at the initial stages of T1D. Absolute counts (cells/µL) of (A) monocytes and their subsets (B) classical and (C) non-classical, and of (D) dendritic cells (DCs) and their subsets (E) myeloid (mDCs) and (F) plasmacytoid (pDCs) were determined in peripheral blood of control subjects and patients with T1D at different time-points. Squares represent control subjects (n=16-17) (white bar), and patients are represented by light grey dots at disease onset (n=17) (grey bar), white dots during the partial remission (PR) phase (n=11) and for remitter patients at 12 months (12M PR) (n=10) (blue bars), and dark grey dots for non-remitter patients at 8 months (8M no PR) (n=6) and 12 months (12M no PR) (n=6) (pink bars). Bar graphs show mean absolute counts values. Each symbol represents an individual patient. Lines link the same patient throughout the different time-points. *P <0.05, **P <0.01 after mixed effects model with Tukey’s post-hoc test for longitudinal data, Kruskal-Wallis with Dunn's post-hoc test for comparisons between control subjects and the different T1D time-points, or 2-tailed Mann-Whitney test for comparisons between two unpaired groups of data. P ≤0.05 is considered significant.


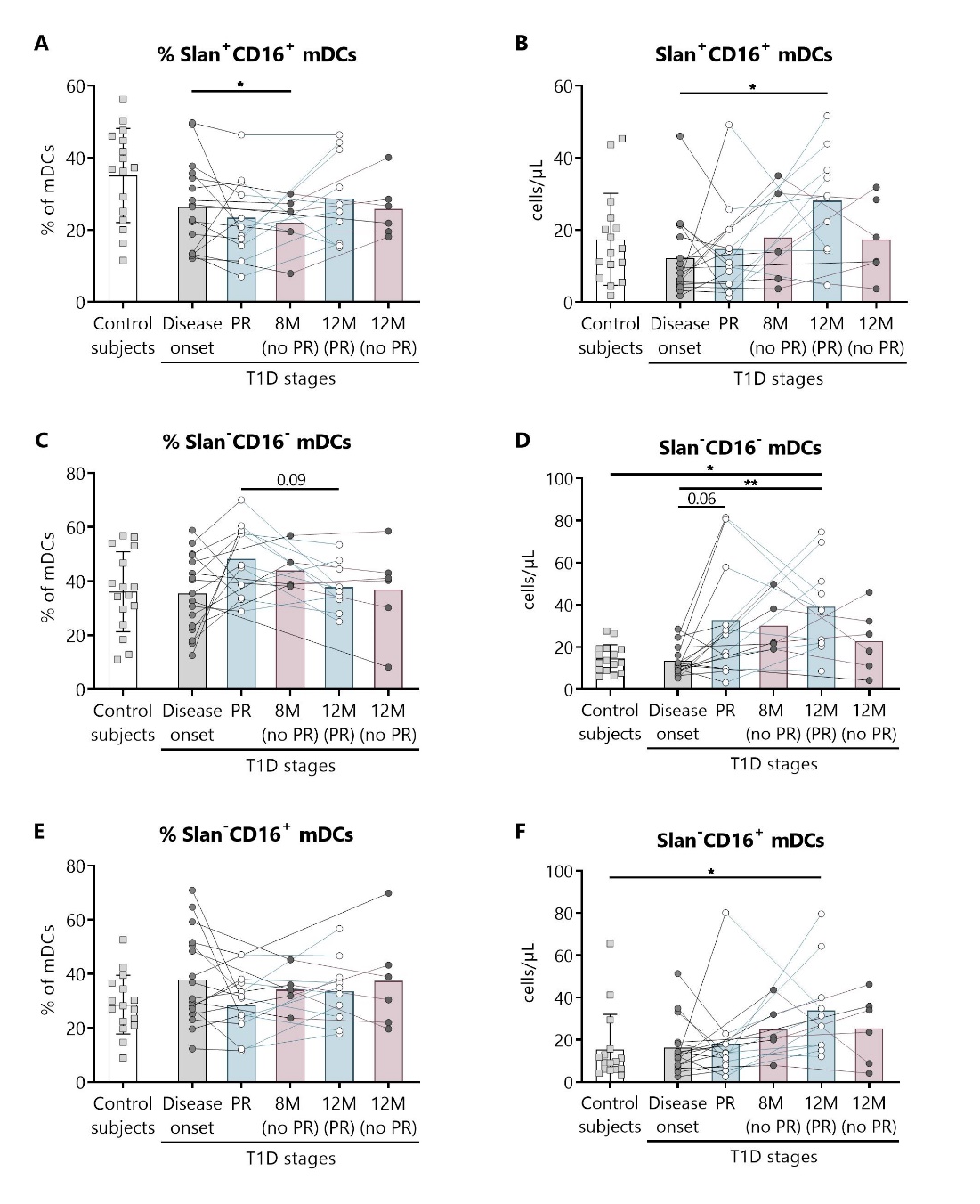
Supplemental Figure 12. Subsets of myeloid dendritic cells defined by the expression of Slan and CD16 are altered at initial stages of T1D. (A, C, E) Percentages (%) and (B, D, F) concentrations (cells/µL) of (A and B) Slan^+^CD16^+^ myeloid dendritic cells (mDCs), (C and D) Slan^-^CD16^-^ mDCs, and (E and F) Slan^-^CD16^+^ mDCs were determined in peripheral blood of control subjects and patients with T1D at different time-points. Squares represent control subjects (n=16) (white bar), and patients are represented by light grey dots at disease onset (n=17) (grey bar), white dots during the partial remission (PR) phase (n=11) and for remitter patients at 12 months (12M PR) (n=10) (blue bars), and dark grey dots for non-remitter patients at 8 months (8M no PR) (n=5) and 12 months (12M no PR) (n=6) (pink bars). Bar graphs show mean percentage or absolute count values. Each symbol represents an individual patient. Lines link the same patient throughout the different time-points. *P <0.05, **P <0.01 after mixed effects model with Tukey’s post-hoc test for longitudinal data, or Kruskal-Wallis with Dunn's post-hoc test for comparisons between control subjects and the different T1D time-points. P ≤0.05 is considered significant.

Supplemental Table 1. Specific markers for phenotypic characterization of leukocyte subpopulations

| Immune cell subsets | Phenotype |
| --- | --- |
| T lymphocytes | |
| Panel 1: T lymphocyte maturation stages | |
| CD4^+^ T lymphocytes | CD3^+^CD4^+^ |
| Naïve CD4^+^ T lymphocytes | CD3^+^CD4^+^CCR7^+^CD45RA^+^ |
| RTEs | CD3^+^CD4^+^CCR7^+^CD45RA^+^CD27^+^CD31^+^PTK7^+^ |
| CM CD4^+^ T lymphocytes | CD3^+^CD4^+^CCR7^+^CD45RA^-^ |
| EM CD4^+^ T lymphocytes | CD3^+^CD4^+^CCR7^-^CD45RA^-^ |
| Late EM CD4^+^ T lymphocytes | CD3^+^CD4^+^CCR7^-^CD45RA^-^CD27^-^ |
| Early EM CD4^+^ T lymphocytes | CD3^+^CD4^+^CCR7^-^CD45RA^-^CD27^+^ |
| CD4^+^ T_EMRA_ lymphocytes | CD3^+^CD4^+^CCR7^-^CD45RA^+^ |
| CD8^+^ T lymphocytes | CD3^+^CD8^+^ |
| Naïve CD8^+^ T lymphocytes | CD3^+^CD8^+^CCR7^+^CD45RA^+^ |
| CM CD8^+^ T lymphocytes | CD3^+^CD8^+^CCR7^+^CD45RA^-^ |
| EM CD8^+^ T lymphocytes | CD3^+^CD8^+^CCR7^-^CD45RA^-^ |
| Late EM CD8^+^ T lymphocytes | CD3^+^CD8^+^CCR7^-^CD45RA^-^CD27^-^ |
| Early EM CD8^+^ T lymphocytes | CD3^+^CD8^+^CCR7^-^CD45RA^-^CD27^+^ |
| CD8^+^ T_EMRA_ lymphocytes | CD3^+^CD8^+^CCR7^-^CD45RA^+^ |
| Panel 2: T_REG_ | |
| CD127^-^CD25^+^ T_REG_ | CD45^+^CD3^+^CD4^+^CD127^-/low^CD25^hi^ |
| Memory T_REG_ | CD45^+^CD3^+^CD4^+^CD127^-/low^CD25^hi^CD45RO^+^CCR4^+^ |
| Activated (HLA-DR^+^) T_REG_ | CD45^+^CD3^+^CD4^+^CD127^-/low^CD25^hi^CD45RO^+^CCR4^+^HLA-DR^+^ |
| Panel 3: T_H_17 lymphocytes | |
| T_H_17 | CD4^+^CCR7^-^CCR4^+^CCR6^+^ |
| Panel 4: TCR | |
| αβ TCR cells | CD3^+^αβ^+^ |
| αβ TCR CD4^+^ cells | CD3^+^αβ^+^CD4^+^ |
| αβ TCR CD8^+^ cells | CD3^+^αβ^+^CD8^+^ |
| αβ TCR DP cells | CD3^+^αβ^+^CD4^+^CD8^+^ |
| αβ TCR DN cells | CD3^+^αβ^+^CD4^-^CD8^-^ |
| γδ TCR cells | CD3^+^γδ^+^ |
| γδ TCR CD4^+^ cells | CD3^+^γδ^+^CD4^+^ |
| γδ TCR CD8^+^ cells | CD3^+^γδ^+^CD8^+^ |
| γδ TCR DP cells | CD3^+^γδ^+^CD4^+^CD8^+^ |
| γδ TCR DN cells | CD3^+^γδ^+^CD4^-^CD8^-^ |
| B lymphocytes | |
| Panel 5: B lymphocyte maturation stages | |
| B lymphocytes | CD3^-^CD19^+^ |
| Naïve B lymphocytes | CD3^-^CD19^+^CD27^-^IgD^+^IgM^+^ |
| CD21^-/low^ naïve B lymphocytes | CD3^-^CD19^+^CD27^-^IgD^+^IgM^+^CD21^low^ |
| Mature naïve B lymphocytes | CD3^-^CD19^+^CD27^-^IgD^+^IgM^+^CD21^+^ |
| Exhausted memory B lymphocytes | CD3^-^CD19^+^CD27^-^IgD^-^IgM^-^ |
| Unswitched memory B lymphocytes | CD3^-^CD19^+^CD27^+^IgD^+^IgM^+^ |
| IgM memory B lymphocytes | CD3^-^CD19^+^CD27^+^IgD^-^IgM^+^ |
| Switched memory B lymphocytes | CD3^-^CD19^+^CD27^+^IgD^-^IgM^-^ |
| Panel 6: B lymphocyte subpopulations | |
| Plasmablasts | CD19^+^CD27^hi^CD38^hi^ |
| B_REG_ lymphocytes | CD19^+^CD27^+^CD24^hi^ |
| Total transitional B lymphocytes | CD19^+^CD27^-^CD38^hi^CD24^hi^ |
| Transitional T1 B lymphocytes | CD19^+^CD27^-^CD38^hi^CD24^hi^ |
| Transitional T2 B lymphocytes | CD19^+^CD27^-^CD38^hihi^CD24^hihi^ |
| Neutrophils, monocytes, and DCs | |
| Panel 7: Innate cells | |
| Neutrophils | CD45^+^CD3^-^CD19^-^CD14^-^CD56^-^Slan^-^CD16^hi^ |
| Monocytes | CD45^+^CD3^-^CD19^-^CD14^+^ |
| Non-classical CD16^+^ monocytes | CD45^+^CD3^-^CD19^-^CD14^+^CD16^+^ |
| Classical monocytes | CD45^+^CD3^-^CD19^-^CD14^+^CD16^-^ |
| DCs | CD45^+^CD3^-^CD19^-^CD14^-^CD56^-^HLA-DR^+^ |
| pDCs | CD45^+^CD3^-^CD19^-^CD14^-^CD56^-^HLA-DR^+^CD123^+^CD11c^-^ |
| mDCs | CD45^+^CD3^-^CD19^-^CD14^-^CD56^-^HLA-DR^+^CD123^-^CD11c^+^ |
| Slan^-^CD16^-^ mDCs | CD45^+^CD3^-^CD19^-^CD14^-^CD56^-^HLA-DR^+^CD123^-^CD11c^+^slan^-^CD16^-^ |
| Slan^-^CD16^+^ mDCs | CD45^+^CD3^-^CD19^-^CD14^-^CD56^-^HLA-DR^+^CD123^-^CD11c^+^slan^-^CD16^+^ |
| Slan^+^CD16^+^ mDCs | CD45^+^CD3^-^CD19^-^CD14^-^CD56^-^HLA-DR^+^CD123^-^CD11c^+^slan^+^CD16^+^ |

HLA-DR, human leukocyte antigen DR; RTEs, recent thymic emigrants; CM, central memory; EM, effector memory; T_EMRA_, T effector memory CD45RA^+^; TCR, T-cell receptor; DP, double-positive; DN, double negative; B_reg_, B regulatory; DCs, dendritic cells; pDCs, plasmacytoid DCs; mDCs, myeloid DCs; Slan, 6-sulfo LacNac.

Supplemental Table 2. A description of all antibodies used in this study

|  | Target | Fluorochrome | Species/Isotype | Clone | Catalog # | Company |
| --- | --- | --- | --- | --- | --- | --- |
| PANEL 1 | CD3 | Violet 500 | Mouse BALB/c IgG_1_, κ | UCHT1 | 561417 | BD Biosciences |
|  | CD4 | PerCP-Cy5.5 | Mouse IgG_1_, κ | RPA-T4 | 560650 | BD Biosciences |
|  | CD8 | APC-H7 | Mouse BALB/c IgG_1_, κ | SK1 | 561423 | BD Biosciences |
|  | CD27 | Brilliant Violet 421 | Mouse IgG_1_, κ | 0323 | 302823 | BioLegend |
|  | CD45RA | FITC | Mouse BALB/c IgG_1_, κ | L48 | 347723 | BD Biosciences |
|  | CCR7 | PE-Cy7 | Rat IgG_2a_, κ | 3D12 | 560922 | BD Biosciences |
|  | CD31 | Alexa Fluor 647 | Mouse IgG_2a_ | M89D3 | 558094 | BD Biosciences |
|  | PTK7 | PE | Mouse IgG_2a_, κ | 188B | 130-099-109 | Miltenyi Biotec |
| PANEL 2 | CD45 | FITC | Mouse IgG_1_, κ | HI30 | 555482 | BD Biosciences |
|  | CD3 | Violet 450 | Mouse BALB/c IgG_1_, κ | UCHT1 | 560365 | BD Biosciences |
|  | CD4 | PerCP-Cy5.5 | Mouse IgG_1_, κ | RPA-T4 | 560650 | BD Biosciences |
|  | CD25 | PE | Mouse BALB/c IgG_1_, κ | M-A251 | 557138 | BD Biosciences |
|  | CD127 | Alexa Fluor 647 | Mouse IgG_1_, κ | HIL-7R-M21 | 558598 | BD Biosciences |
|  | CD45RO | APC-H7 | Mouse BALB/c IgG_2a_, κ | UCHL1 | 561137 | BD Biosciences |
|  | CCR4 | PE-Cy7 | Mouse C57BL/6 IgG_1_, κ | 1G1 | 561034 | BD Biosciences |
|  | HLA-DR | Violet 500 | Mouse IgG_2a_, κ | G46-6 | 561225 | BD Biosciences |
| PANEL 3 | CD4 | Violet 450 | Mouse IgG_1_, κ | RPA-T4 | 561838 | BD Biosciences |
|  | CCR7 | PE-Cy7 | Rat IgG_2a_, κ | 3D12 | 560922 | BD Biosciences |
|  | CCR4 | Alexa Fluor 647 | Mouse C57BL/6 IgG_1_, κ | 1G1 | 557863 | BD Biosciences |
|  | CCR6 | PE | Mouse IgG_1_, κ | 11A9 | 551773 | BD Biosciences |
| PANEL 4 | CD3 | PerCP | Mouse BALB/c IgG_1_, κ | SK7 | 347344 | BD Biosciences |
|  | CD4 | Violet 450 | Mouse IgG_1_, κ | RPA-T4 | 561838 | BD Biosciences |
|  | CD8 | APC-H7 | Mouse BALB/c IgG_1_, κ | SK1 | 561423 | BD Biosciences |
|  | αβ TCR | FITC | Mouse BALB/c IgM, κ | T10B9 | 555547 | BD Biosciences |
|  | γδ TCR | PE | Mouse IgG_1_, κ | B1 | 561994 | BD Biosciences |
| PANEL 5 | CD3 | Violet 450 | Mouse BALB/c IgG_1_, κ | UCHT1 | 560365 | BD Biosciences |
|  | CD19 | Violet 500 | Mouse IgG_1_, κ | HIB19 | 561125 | BD Biosciences |
|  | CD27 | APC | Mouse BALB/c IgG_1_ | L128 | 337169 | BD Biosciences |
|  | IgD | FITC | Mouse BALB/c IgG_2a_, κ | IA6-2 | 562023 | BD Biosciences |
|  | IgM | PerCP-Cy5.5 | Mouse IgG_1_, κ | G20-127 | 561285 | BD Biosciences |
|  | CD21 | PE | Mouse IgG_1_, κ | B-ly4 | 561768 | BD Biosciences |
| PANEL 6 | CD19 | PerCP-Cy5.5 | Mouse IgG_1_, κ | HIB19 | 561295 | BD Biosciences |
|  | CD24 | FITC | Mouse IgG_2a_, κ | ML5 | 560992 | BD Biosciences |
|  | CD38 | PE | Mouse IgG_1_, κ | HIT2 | 555460 | BD Biosciences |
|  | CD27 | APC | Mouse BALB/c IgG_1_ | L128 | 337169 | BD Biosciences |
| PANEL 7 | CD45 | Alexa Fluor 700 | Mouse IgG_1_, κ | HI30 | 304023 | BioLegend |
|  | CD19 | APC-H7 | Mouse IgG_1_, κ | HIB19 | 560727 | BD Biosciences |
|  | CD3 | APC-H7 | Mouse BALB/c IgG_1_, κ | SK7 | 560176 | BD Biosciences |
|  | CD14 | Violet 450 | Mouse BALB/c IgG_2b_, κ | MφP9 | 560349 | BD Biosciences |
|  | CD16 | APC | Mouse BALB/c IgG_1_, κ | B73.1 | 561306 | BD Biosciences |
|  | Slan | FITC | Mouse IgM, κ | DD-1 | 130-117-520 | Miltenyi Biotec |
|  | HLA-DR | Violet 500 | Mouse IgG2a, κ | G46-6 | 561225 | BD Biosciences |
|  | CD11c | PE-Cy7 | Mouse BALB/c IgG_1_, κ | B-ly6 | 561356 | BD Biosciences |
|  | CD123 | PerCP-Cy5.5 | Mouse IgG_2a_, κ | 7G3 | 560904 | BD Biosciences |
|  | CD56 | PE | Mouse IgG_1_, κ | MY31 | 556647 | BD Biosciences |

PerCP-Cy5.5, peridinin-chlorophyll-cyanine 5.5; APC-H7, allophycocyanin-hilite 7; FITC, fluorescein isothiocyanate; PE-Cy7, phycoerythrin-cyanine 7; PE, phycoerythrin; APC, allophycocyanin; PerCP, peridinin-chlorophyll-protein.

Supplemental Table 3. Clinical features and metabolic data of remitter patients classified regarding their decreasing (PR) or increasing (non-PR-like) percentages of T_REG_ cells from baseline.

|  | PR (n=7) | PR (non-PR-like) (n=4) | P value |
| --- | --- | --- | --- |
| Age (years) | 11.1 ± 3.98 | 5.5 ± 1.3 | 0.01* |
| Sex (M/F) | 1/6 | 4/0 | 0.006** |
| BMI (kg/m2) | 18.97 ± 3 | 15.6 ± 1.6 | 0.02* |
| BMI-SDS | -0.1 (-0.6, 1.1) | -0.4 (-1.2, 0.65) | 0.53 |
| HbA1c (%) | 6.6 ± 0.6 | 7.3 ± 0.45 | 0.12 |
| HbA1c (mmol/mol) | 49 ± 6.1 | 55.75 ± 4.8 | 0.61 |
| Insulin dose (U/Kg/day) | 0.44 ± 0.1 | 0.28 ± 0.01 | 0.02* |
| Basal C-peptide (ng/mL) | 1.03 ± 0.5 | 0.28 ± 0.05 | 0.03* |

Data presented as mean ± SD, or mean (min, max). *P* value calculated from Mann-Whitney test (**P* ≤0.05 and ***P* ≤0.01). BMI, Body Mass Index; F, female; HbA1c, glycated hemoglobin; IDAA1c, insulin dose-adjusted HbA1c; M, male; SDS, standard deviation score.

Supplemental Table 4. Linear regression of covariates at type 1 diabetes onset

| Effect | DFn^D^ | DFd^E^ | F value^F^ | P value^G^ |
| --- | --- | --- | --- | --- |
| Age (years)^A^ | 1 | 23 | 1.714 | 0.2035 |
| Age (years)^B^ | 1 | 22 | 0.7233 | 0.4042 |
| Age (years)^C^ | 1 | 22 | 0.06134 | 0.8067 |
| Sex (M/F)^A^ | 1 | 23 | 0.2592 | 0.6155 |
| Sex (M/F)^B^ | 1 | 22 | 1.341 | 0.2593 |
| Sex (M/F)^C^ | 1 | 22 | 1.124 | 0.3006 |
| BMI (kg/m^2^)^A^ | 1 | 23 | 1.429 | 0.2441 |
| BMI (kg/m^2^)^B^ | 1 | 22 | 0.9013 | 0.3528 |
| BMI (kg/m^2^)^C^ | 1 | 22 | 0.009854 | 0.9218 |
| BMI-SDS^A^ | 1 | 23 | 0.5799 | 0.4541 |
| BMI-SDS^B^ | 1 | 22 | 2.117 | 0.1598 |
| BMI-SDS^C^ | 1 | 22 | 0.003669 | 0.9522 |
| HbA1c (%)^A^ | 1 | 23 | 0.00359 | 0.9527 |
| HbA1c (%)^B^ | 1 | 22 | 2.271 | 0.1460 |
| HbA1c (%)^C^ | 1 | 22 | 0.2332 | 0.6339 |
| Basal C-peptide (ng/mL)^A^ | 1 | 23 | 1.372 | 0.2535 |
| Basal C-peptide (ng/mL)^B^ | 1 | 22 | 0.4639 | 0.5029 |
| Basal C-peptide (ng/mL)^C^ | 1 | 22 | 0.04781 | 0.8289 |
| Stimulated C-peptide (ng/mL)^A^ | 1 | 20 | 0.6909 | 0.4157 |
| Stimulated C-peptide (ng/mL)^B^ | 1 | 19 | 0.09984 | 0.7555 |
| Stimulated C-peptide (ng/mL)^C^ | 1 | 19 | 0.04853 | 0.8280 |
| IDAA1c^A^ | 1 | 23 | 0.09683 | 0.7585 |
| IDAA1c^B^ | 1 | 22 | 2.705 | 0.1142 |
| IDAA1c^C^ | 1 | 22 | 0.068 | 0.7967 |
| Insulin dose (U/kg/day)^A^ | 1 | 23 | 4.159 | 0.0531 |
| Insulin dose (U/kg/day)^B^ | 1 | 22 | 0.5758 | 0.4560 |
| Insulin dose (U/kg/day)^C^ | 1 | 22 | 0.4028 | 0.5322 |

^A^Parameters tested as effectors of T_REG_ percentage. ^B^Parameters tested as effectors of DC percentage. ^C^Parameters tested as effectors of Monocyte cell percentage. ^D^The numbers of degrees of freedom. ^E^The denominator degrees of freedom. ^F^The F value is the result of the test where the null hypothesis is that all of regression coefficients are equal to 0. ^G^The null hypothesis is that the predictor has no effect on the outcome variable evaluated regarding this P value. BMI, Body Mass Index; HbA1c, glycated hemoglobin; IDAA1c, insulin dose-adjusted HbA1c; SDS, standard deviation score.

Supplemental Table 5. Simple logistic regressions for immune cell determinants of partial remission in pediatric patients at type 1 diabetes onset

| Simple logistic regressions | | | | | | | | |
| --- | --- | --- | --- | --- | --- | --- | --- | --- |
| Variable | Coefficient | SE | OR | 95% CI for OR | \|Z\| | P value^A^ | G test | P value^B^ |
| Immunological variables | | | | | | | | |
| Peripheral immune cells (%) | | | | | | | | |
| CD4^+^ T lymphocytes | -0.035 | 0.050 | 0.97 | 0.87 to 1.06 | 0.711 | 0.48 | 0.519 | 0.47 |
| Memory T_REG_ | -0.121 | 0.458 | 0.89 | 0.35 to 2.26 | 0.265 | 0.79 | 0.070 | 0.79 |
| Activated (HLA-DR^+^) T_REG_ | 0.058 | 0.059 | 1.06 | 0.95 to 1.21 | 0.972 | 0.33 | 1.034 | 0.31 |
| Naïve CD4^+^ T lymphocytes | 0.015 | 0.050 | 1.02 | 0.92 to 1.12 | 0.292 | 0.77 | 0.085 | 0.77 |
| RTEs | -0.086 | 0.105 | 0.92 | 0.73 to 1.13 | 0.815 | 0.42 | 0.687 | 0.41 |
| CM CD4^+^ T lymphocytes | 0.008 | 0.071 | 1.01 | 0.88 to 1.17 | 0.117 | 0.91 | 0.014 | 0.91 |
| EM CD4^+^ T lymphocytes | 0.045 | 0.062 | 1.05 | 0.93 to 1.20 | 0.730 | 0.47 | 0.559 | 0.45 |
| Late EM CD4^+^ T lymphocytes | 0.043 | 0.128 | 1.04 | 0.82 to 1.44 | 0.333 | 0.74 | 0.116 | 0.73 |
| Early EM CD4^+^ T lymphocytes | 0.046 | 0.070 | 1.05 | 0.92 to 1.22 | 0.660 | 0.51 | 0.454 | 0.50 |
| CD4^+^ T_EMRA_ lymphocytes | -0.044 | 0.047 | 0.96 | 0.86 to 1.05 | 0.925 | 0.36 | 0.882 | 0.35 |
| T_H_17 | 0.049 | 0.160 | 1.05 | 0.77 to 1.48 | 0.307 | 0.76 | 0.095 | 0.76 |
| CD8^+^ T lymphocytes | -0.002 | 0.080 | 1.00 | 0.85 to 1.17 | 0.021 | 0.98 | 0.0004 | 0.98 |
| Naïve CD8^+^ T lymphocytes | -0.027 | 0.033 | 0.97 | 0.91 to 1.04 | 0.823 | 0.41 | 0.695 | 0.40 |
| CM CD8^+^ T lymphocytes | -0.139 | 0.134 | 0.87 | 0.65 to 1.14 | 1.011 | 0.31 | 1.047 | 0.31 |
| EM CD8^+^ T lymphocytes | 0.012 | 0.046 | 1.01 | 0.92 to 1.11 | 0.254 | 0.80 | 0.064 | 0.80 |
| Late EM CD8^+^ T lymphocytes | -0.119 | 0.114 | 0.89 | 0.64 to 1.05 | 1.036 | 0.30 | 1.841 | 0.17 |
| Early EM CD8^+^ T lymphocytes | 0.081 | 0.063 | 1.08 | 0.97 to 1.24 | 1.296 | 0.20 | 1.855 | 0.17 |
| CD8^+^ T_EMRA_ lymphocytes | 0.065 | 0.055 | 1.07 | 0.97 to 1.20 | 1.200 | 0.23 | 1.574 | 0.21 |
| αβ TCR cells | -0.157 | 0.098 | 0.85 | 0.66 to 0.99 | 1.595 | 0.11 | 4.816 | 0.03* |
| αβ TCR CD4^+^ cells | 0.026 | 0.093 | 1.03 | 0.86 to 1.26 | 0.284 | 0.777 | 0.082 | 0.77 |
| αβ TCR CD8^+^ cells | -0.031 | 0.094 | 0.97 | 0.79 to 1.16 | 0.334 | 0.74 | 0.114 | 0.74 |
| αβ TCR DP cells | 0.158 | 0.61 | 1.17 | 0.40 to 7.82 | 0.259 | 0.80 | 0.073 | 0.79 |
| αβ TCR DN cells | -0.153 | 0.860 | 0.859 | 0.15 to 5.36 | 0.177 | 0.86 | 0.031 | 0.86 |
| γδ TCR cells | 0.471 | 0.248 | 1.60 | 1.05 to 2.88 | 1.897 | 0.06 | 4.868 | 0.03* |
| γδ TCR CD4^+^ cells | -0.478 | 0.312 | 0.62 | 0.27 to 0.95 | 1.532 | 0.13 | 5.242 | 0.02* |
| γδ TCR CD8^+^ cells | -0.061 | 0.056 | 0.94 | 0.84 to 1.05 | 1.091 | 0.28 | 1.232 | 0.27 |
| γδ TCR DP cells | -4.752 | 2.853 | 0.009 | 3.06e^-006^ to 0.81 | 1.666 | 0.10 | 4.257 | 0.04* |
| γδ TCR DN cells | 0.107 | 0.061 | 1.11 | 1.00 to 1.28 | 1.756 | 0.08 | 3.621 | 0.06 |
| B lymphocytes | -0.070 | 0.097 | 0.93 | 0.76 to 1.13 | 0.727 | 0.47 | 0.538 | 0.46 |
| Naïve B lymphocytes | -0.015 | 0.031 | 0.98 | 0.92 to 1.05 | 0.506 | 0.61 | 0.259 | 0.61 |
| CD21^-/low^ naïve B lymphocytes | 0.031 | 0.122 | 1.03 | 0.82 to 1.34 | 0.255 | 0.80 | 0.066 | 0.80 |
| Mature naïve B lymphocytes | -0.011 | 0.114 | 0.99 | 0.78 to 1.24 | 0.096 | 0.92 | 0.009 | 0.92 |
| Exhausted memory B lymphocytes | 0.013 | 0.090 | 1.01 | 0.85 to 1.25 | 0.149 | 0.88 | 0.022 | 0.88 |
| Unswitched memory B lymphocytes | 0.024 | 0.081 | 1.03 | 0.88 to 1.22 | 0.302 | 0.76 | 0.092 | 0.76 |
| IgM memory B lymphocytes | -0.627 | 0.762 | 0.53 | 0.10 to 2.33 | 0.823 | 0.41 | 0.700 | 0.40 |
| Switched memory B lymphocytes | 0.040 | 0.071 | 1.04 | 0.91 to 1.23 | 0.560 | 0.58 | 0.339 | 0.56 |
| Plasmablasts | 0.106 | 0.183 | 1.11 | 0.90 to 2.44 | 0.580 | 0.56 | 0.665 | 0.41 |
| B_REG_ lymphocytes | -0.0003 | 0.066 | 1.0 | 0.88 to 1.16 | 0.005 | 0.99 | 2.455e^-005^ | 0.99 |
| Total transitional B lymphocytes | -0.164 | 0.105 | 0.85 | 0.67 to 1.03 | 1.557 | 0.12 | 2.792 | 0.09 |
| Transitional T1 B lymphocytes | -0.021 | 0.047 | 0.98 | 0.89 to 1.10 | 0.442 | 0.66 | 0.198 | 0.66 |
| Transitional T2 B lymphocytes | 0.015 | 0.050 | 1.02 | 0.92 to 1.13 | 0.293 | 0.77 | 0.086 | 0.77 |
| Neutrophils | -0.542 | 0.642 | 0.58 | 0.13 to 1.01 | 0.844 | 0.40 | 1.669 | 0.20 |
| Non-classical CD16^+^ monocytes | -0.119 | 0.108 | 0.89 | 0.70 to 1.10 | 1.101 | 0.27 | 1.259 | 0.26 |
| Classical monocytes | 0.120 | 0.110 | 1.13 | 0.91 to 1.44 | 1.091 | 0.28 | 1.233 | 0.27 |
| pDCs | -0.074 | 0.126 | 0.93 | 0.71 to 1.19 | 0.586 | 0.56 | 0.349 | 0.55 |
| mDCs | -0.034 | 0.055 | 0.97 | 0.86 to 1.07 | 0.631 | 0.53 | 0.409 | 0.52 |
| Slan^-^CD16^-^ mDCs | -0.011 | 0.029 | 0.99 | 0.93 to 1.05 | 0.386 | 0.70 | 0.150 | 0.70 |
| Slan^-^CD16^+^ mDCs | -0.0004 | 0.025 | 1.00 | 0.95 to 1.05 | 0.014 | 0.99 | 0.0002 | 0.99 |
| Slan^+^CD16^+^ mDCs | 0.021 | 0.039 | 1.02 | 0.95 to 1.11 | 0.541 | 0.59 | 0.309 | 0.58 |

^A^P value from OR; ^B^P value from G test. SE, standard error; HLA-DR, human leukocyte antigen DR; RTEs, recent thymic emigrants; CM, central memory; EM, effector memory; T_EMRA_, T effector memory CD45RA^+^; TCR, T-cell receptor; DP, double-positive; DN, double negative; B_reg_, B regulatory; DCs, dendritic cells; pDCs, plasmacytoid DCs; mDCs, myeloid DCs; Slan, 6-sulfo LacNac.

Supplemental Table 6. Collinearity statistics of the multiple regression model

| Multicollinearity | | |
| --- | --- | --- |
| Variable | VIF^A^ | R^2^ with other variables^B^ |
| Index^C^ | 1.002 | 0.0017 |
| BMI-SDS | 1.002 | 0.0017 |

^A^VIF = variance inflation factor; a high VIF (>4) indicates that the associated independent variable is highly collinear with the other variables in the model. ^B^The fraction of the variance in one X variable that can be predicted from the other X variables. ^C^T_REG_ (%) + Monocytes (%) + DCs (%) cut-off values from independent ROC curves as conditions to be fulfilled on a 0-3 scale. BMI, Body Mass Index; SDS; standard deviation score.
